# Peptidoglycan Remodeling Enables Escherichia coli To Survive Severe Outer Membrane Assembly Defect

**DOI:** 10.1128/mBio.02729-18

**Published:** 2019-02-05

**Authors:** Niccolò Morè, Alessandra M. Martorana, Jacob Biboy, Christian Otten, Matthias Winkle, Carlos K. Gurnani Serrano, Alejandro Montón Silva, Lisa Atkinson, Hamish Yau, Eefjan Breukink, Tanneke den Blaauwen, Waldemar Vollmer, Alessandra Polissi

**Affiliations:** a Dipartimento di Scienze Farmacologiche e Biomolecolari, Università degli Studi di Milano, Milan, Italy; b The Centre for Bacterial Cell Biology, Institute for Cell and Molecular Biosciences, Newcastle University, Newcastle upon Tyne, United Kingdom; c Bacterial Cell Biology & Physiology, Swammerdam Institute for Life Sciences, University of Amsterdam, Amsterdam, The Netherlands; d Membrane Biochemistry and Biophysics, Department of Chemistry, Faculty of Science, Utrecht University, Utrecht, The Netherlands; Nanyang Technological University; Virginia Tech; Institut Pasteur; Queen's University Belfast

**Keywords:** *Escherichia coli*, cell envelope, lipopolysaccharide, peptidoglycan, stress response

## Abstract

Gram-negative bacteria have a tripartite cell envelope with the cytoplasmic membrane (CM), a stress-bearing peptidoglycan (PG) layer, and the asymmetric outer membrane (OM) containing lipopolysaccharide (LPS) in the outer leaflet. Cells must tightly coordinate the growth of their complex envelope to maintain cellular integrity and OM permeability barrier function. The biogenesis of PG and LPS relies on specialized macromolecular complexes that span the entire envelope. In this work, we show that Escherichia coli cells are capable of avoiding lysis when the transport of LPS to the OM is compromised, by utilizing LD-transpeptidases (LDTs) to generate 3-3 cross-links in the PG. This PG remodeling program relies mainly on the activities of the stress response LDT, LdtD, together with the major PG synthase PBP1B, its cognate activator LpoB, and the carboxypeptidase PBP6a. Our data support a model according to which these proteins cooperate to strengthen the PG in response to defective OM synthesis.

## Abstract (precis)

In Gram-negative bacteria, the outer membrane protects the cell against many toxic molecules, and the peptidoglycan layer provides protection against osmotic challenges, allowing bacterial cells to survive in changing environments. Maintaining cell envelope integrity is therefore a question of life or death for a bacterial cell. Here we show that Escherichia coli cells activate the LD-transpeptidase LdtD to introduce 3-3 cross-links in the peptidoglycan layer when the integrity of the outer membrane is compromised, and this response is required to avoid cell lysis. This peptidoglycan remodeling program is a strategy to increase the overall robustness of the bacterial cell envelope in response to defects in the outer membrane.

## INTRODUCTION

The integrity of a diderm (Gram-negative) bacterial cell is maintained by a complex cell envelope composed of the cytoplasmic membrane (CM), the periplasm with a thin peptidoglycan (PG) sacculus, and the outer membrane (OM) (1, 2). The asymmetrical OM contains in the outer leaflet lipopolysaccharide (LPS) (3), which makes the cell envelope impermeable to many toxic compounds and antibiotics (4).

LPS is assembled at the outer leaflet of the CM (5–7) and then transported across the periplasm to reach its final destination at the outermost surface of the cell (8, 9). In Escherichia coli, LPS transport is facilitated by seven essential proteins, LptA to LptG (10–15) which form a transenvelope protein bridge through the periplasm and its PG sacculus (11, 16–18). This organization allows the coupling of ATP hydrolysis with LPS movement across the periplasm up to the cell surface, as proposed in the so-called PEZ model (19). Depletion of any of the Lpt components results in block of LPS transport and its accumulation at the periplasmic leaflet of the cytoplasmic membrane (CM) (12, 14).

LPS export to the OM is among the cell's main transport processes. Considering a generation time of 20 min for fast growing E. coli, LPS transport must occur at a rate of more than 10^3^ molecules per second to ensure complete coverage of the cell surface during growth (20). Moreover, the supply of LPS must be optimally coupled to the synthesis and assembly of other cell envelope components, such as PG, to prevent loss of OM integrity due to LPS depletion or detrimental effects by excessive LPS production. How LPS synthesis and export is regulated remains largely unknown.

LPS is exported through the periplasmic PG sacculus that has a net-like structure composed of glycan strands connected by short, cross-linked peptides (2, 21). PBP1A and PBP1B are major and semiredundant PG synthases that polymerize glycan strands by their glycosyltransferase (GTase) activity and cross-link stem peptides by DD-transpeptidase (DD-TPase) activity, forming the abundant 4-3 cross-links in PG (see Fig. S1A in the supplemental material) (22–24). The CM-anchored PBPs require activation by their cognate, OM-anchored lipoprotein (LpoA and LpoB, respectively) (25–27). LpoA and LpoB span the periplasm to activate their cognate PBP (28–30), presumably responding to the size of pores in the PG layer to couple PG growth with cell growth (21).

10.1128/mBio.02729-18.1 FIG S1 Peptidoglycan cross-linking reactions and sequence alignment of the LdtD, LdtE, and LdtF proteins. Download FIG S1, PDF file, 0.2 MB. Copyright © 2019 Morè et al. 2019 Morè et al. This content is distributed under the terms of the Creative Commons Attribution 4.0 International license.

DD-carboxypeptidases (DD-CPase) such as PBP5, PBP6a, and PBP6b trim the pentapeptides present in new PG to tetrapeptides (31–33). PBP5 is the major DD-CPase in the cell; its absence causes aberrant cell morphology in strains lacking other PBPs (33, 34). PBP6b contributes substantially to PG remodeling and cell shape maintenance in cells growing at acidic pH (35).

In E. coli, the majority (90% to 98%) of cross-links in PG are of the 4-3 (or DD) type (between D-Ala and *meso*-diaminopimelic acid [*meso*-Dap]) (36). However, there are 2 to 10% of the 3-3 (or LD) type of cross-links between two *meso*-Dap residues of adjacent stem peptides (Fig. S1A), and these increase to up to 16% in stationary-phase cells (36, 37). 3-3 cross-links are produced by LD-transpeptidases (LDTs) of the YkuD family of proteins (PF03734), which are structurally unrelated to PBPs. LDTs use tetrapeptide donors in the TPase reaction and are insensitive to most β-lactams (Fig. S1A) (38).

E. coli has five LDTs with two distinct functions. LdtD (formerly YcbB) and LdtE (YnhG) form 3-3 cross-links, whereas LdtA (ErfK), LdtB (YbiS), and LdtC (YcfS) attach the abundant OM-anchored Lpp (Braun's lipoprotein) to *meso*-Dap residues in PG, providing a tight connection between the PG and OM. Notably, E. coli mutants with multiple or all *ldt* genes deleted exhibit only minor phenotypes, suggesting that these functions are dispensable during growth under laboratory conditions (39–41).

Certain strains of Enterococcus faecium can grow in the presence of β-lactam antibiotics using a β-lactam-insensitive LDT, Ldt_fm_ to produce 3-3 cross-links instead of the β-lactam-sensitive PBP TPases (42–44). More recently, a DD-TPase-independent and LDT-dependent mutant strain of E. coli has been selected by its ability to grow at a high and otherwise lethal concentration of ampicillin, at which it produces exclusively 3-3 cross-links in its PG (45). This strain has an elevated level of the alarmone (p)ppGpp and needs LdtD, the DD-CPase PBP5, and the GTase domain of PBP1B together with its regulator, LpoB, to bypass PBPs and achieve broad-spectrum β-lactam resistance (45). However, E. coli strains do not readily acquire this mechanism of resistance, and it is possible that the 3-3 cross-linking activities of LdtD and LdtE have another, yet undiscovered function in E. coli.

In this work, we show that E. coli cells defective in the LPS export pathway require LDTs that produce an increased level of 3-3 cross-links in the PG to avoid cell lysis. Our data suggest that LdtD is specifically expressed in response to OM damage and participates in a PG remodeling program activated in response to the block of LPS transport. Notably, PG remodeling also involves the GTase activity of PBP1B and the DD-CPase of previously unknown function, PBP6a. We propose a model whereby PBP1B, LdtD, and PBP6a cooperate in a dedicated PG machine which is needed when LPS transport is compromised.

## RESULTS

### Defective LPS export induces the formation of 3-3 cross-links in PG.

We previously observed that several PG-synthesizing or PG-modifying enzymes are upregulated upon depletion of the essential LptC component of the LPS export machinery (46), prompting us to analyze the composition of PG isolated from cells with compromised LPS transport.

**FIG 1:**
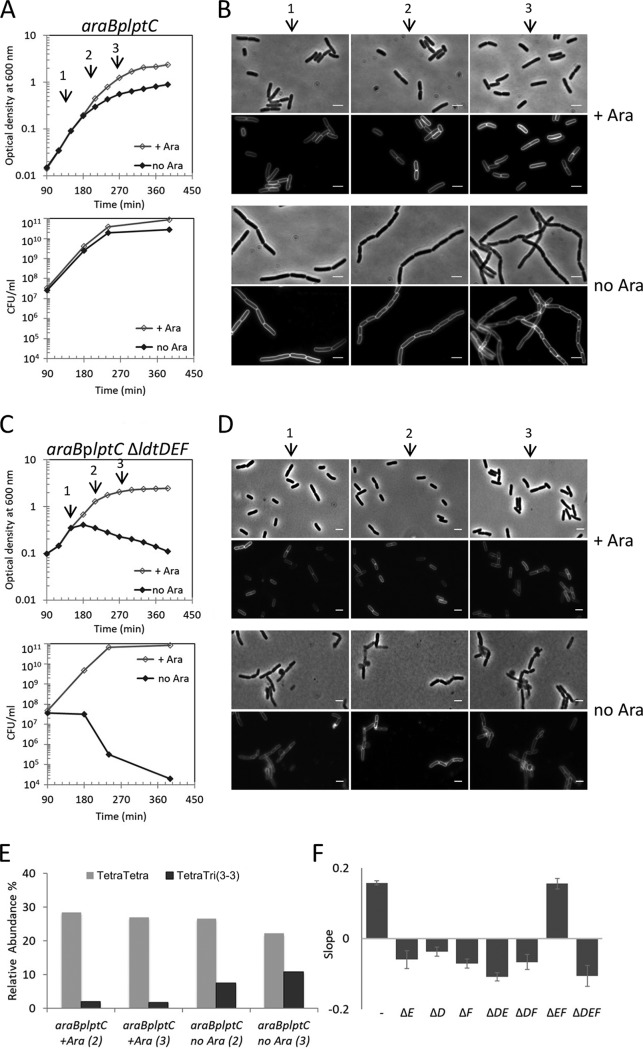
LDTs prevent cell lysis upon defective OM assembly. (A to D) Cells of the *araB*p*lptC* conditional strain (A and B) and the isogenic mutants with *ldtD*, *ldtE*, and *ldtF* deleted (C and D) were grown in the presence of 0.2% arabinose to an OD_600_ of 0.2, harvested, washed three times, and resuspended in an arabinose-supplemented (+ Ara) or arabinose-free (no Ara) medium. (A and C) Growth was monitored by OD_600_ measurements (top panels) and by determining CFU (bottom panels). Growth curves shown are representative of at least three independent experiments. At *t* = 120, 210, and 270 min (arrows), samples were imaged (*araB*p*lptC* [B]; isogenic mutant deleted for *ldtD*, *ldtE*, and *ldtF* [D]). Phase-contrast images (top) and fluorescence images (bottom) are shown. Bars, 3 μm. (E) PG sacculi purified from *araB*p*lptC* cells grown in the presence of arabinose or after 210 min (2) or 270 min (3) growth in the absence of arabinose were digested with cellosyl, and the muropeptide composition was determined by HPLC. The graph shows the relative abundance of TetraTetra (with a 4-3 cross-link) and TetraTri(3-3) (with a 3-3 cross-link) muropeptides. The latter significantly increased upon depletion of LptC. (F) Cells of the *araB*p*lptC* conditional strain and isogenic mutants deleted for every *ldt* gene alone or in all possible combinations were grown in an arabinose-free medium as indicated above. Growth phenotypes are summarized as the slope of growth curves measured between 180 and 390 min. Positive and negative values indicate cell growth and cell lysis, respectively. Values are means plus standard deviations (SD) (error bars) from three independent experiments. The mean slope calculated from growth curves in arabinose-supplemented medium for the *araB*p*lptC* conditional strain and isogenic *ldt* mutants was 0.56 ± 0.03. The *ldt* genes are indicated by their loci shown by capital letters.

For this purpose, we cultured an *araB*p*lptC* conditional strain, in which *lptC* expression is under the control of the arabinose-inducible *araB*p promoter. As previously reported (13), LptC-depleted cells formed short chains and arrested growth (Fig. 1A and B). The sacculi purified from these cells showed a four- to sixfold increase in the relative amount of 3-3 cross-links between two *meso*-Dap residues compared to sacculi from cells grown in the presence of arabinose (Fig. 1E and Table 1; see also Table S3 in the supplemental material). 3-3 cross-links increased early in LptC-depleted cells, indicating a rapid cellular response to the LPS transport arrest. We also observed only a moderate decrease in the canonical 4-3 (*meso*-Dap to D-Ala) cross-links in LptC-depleted cells (Fig. 1E and Table S3).

**TABLE 1:** Summary of the level of 3-3 cross-links in PG and growth phenotype of single and multiple *ldt* mutant strains with or without depletion of LPS export^*a*^

Presence/absence of gene			3-3 cross-linkage or phenotype in:					
			*lptC*^+^ strain, 3-3 CL (area [%])^*b*^	*araB*p*lptC* strain				
				With arabinose		No arabinose		
*ldtD*	*ldtE*	*ldtF*		Growth	3-3 CL (area [%])	Growth	3-3 CL (area [%])	Lysis rescue by p*ldtD*
+	+	+	3.0	Normal	1.7	Arrest	7.5	NT^*e*^
−	+	+	3.2	Normal	2.4	Lysis	6.1	+
+	−	+	2.9	Normal	1.9	Lysis	6.0	+
+	+	−	2.9	Normal	1.9	Lysis	8.4	+
−	−	+	2.2	Normal	1.9	Lysis	−^*d*^	+
−	+	−	ND^*c*^	Normal	ND^*c*^	Lysis	ND	+
+	−	−	2.4	Normal	8.2	Arrest	8.4	NT
−	−	−	ND	Normal	ND	Lysis	ND	+

a The table shows representative data of muropeptide analysis. The details of muropeptide profiles of repeats are shown in Table S3 in the supplemental material.

b Sum of the percentages of all muropeptides with 3-3 cross-links (CL) in the muropeptide profile. See Table S3 for complete data on muropeptide composition.

c ND, not detected. 3-3 cross-linked muropeptides were below the detection limit.

d −, not determined because the LptC-depleted cells lysed rapidly, preventing reliable peptidoglycan analysis.

e NT, not tested because cells do not lyse.

### 3-3 cross-links are not essential under standard growth conditions.

E. coli has five LDTs (LdtA to LdtE) (39–41). When inspecting the E. coli genome, we identified another hypothetical *ldt* gene, *yafK*. The predicted YafK shares 33% and 41% sequence identity to the catalytic YkuD (LDT) domain of LdtD and LdtE, respectively, but lacks a conserved arginine residue near the active site cysteine and might not be fully active (Fig. S1B). We included *yafK* (now termed *ldtF*) in our further studies on the roles of LDTs in the formation of 3-3 cross-links during defective LPS export.

To assess the roles of the LDTs in E. coli, we examined the growth phenotypes and levels of 3-3 cross-links in sacculi purified from all possible single and multiple deletion mutants. The deletion of *ldtD*, *ldtE*, and *ldtF* alone and in all possible combinations did not affect the growth of E. coli (Table 1 and Fig. S3A and B). Even the deletion of all six *ldt* genes did not result in any growth defect under standard laboratory conditions (data not shown). The muropeptide analysis revealed that only 3.0% of the PG muropeptides from strain BW25113 contained 3-3 cross-links (Table 1 and Table S3), consistent with earlier reports (36, 37). The Δ*ldtD* Δ*ldtE* mutant contained 2.2% muropeptides with 3-3 cross-links, and 3-3 cross-links were not detected in the PG from the Δ*ldtD* Δ*ldtE* Δ*ldtF* triple mutant, suggesting that E. coli has no other enzyme for 3-3 cross-link formation in the absence of LdtD, LdtE, and LdtF. The Δ*ldtD* Δ*ldtF* double mutant did not produce detectable levels of 3-3 cross-links, suggesting that LdtE is either not active as an LD-TPase or it requires LdtD and/or LdtF for activity. In all other *ldt* defective strains, the level of 3-3 cross-links was comparable to that of the BW25113 wild-type strain, suggesting that one or more LDTs is active in these mutants (Table 1 and Table S3).

**FIG 2:**
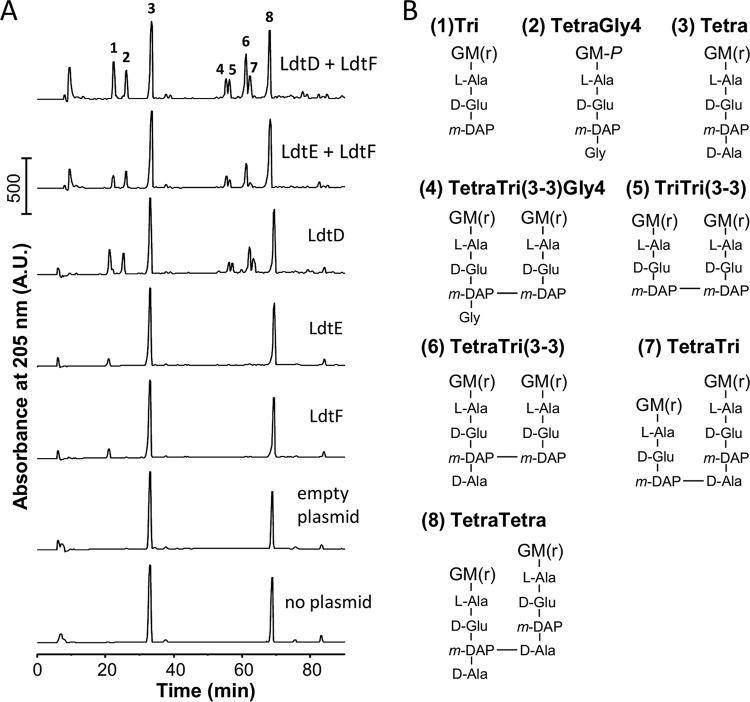
Ectopic expression of LdtD and LdtE-LdtF results in 3-3 cross-links. (A) Muropeptide profiles of *E. coli* BW25113Δ6LDT cells containing either no plasmid, empty plasmid (pJEH12), or plasmid with *ldtD* (pJEH12-*ldtD*), *ldtE* (pAMS01*-ldtE*), *ldtF* (pAMS02*-ldtF*), *ldtE*-*ldtF* (pAMS01*-ldtE* and pGS124), or *ldtD*-*ldtF* (pJEH12-*ldtD* and pGS124) grown in the presence of inducer. A.U., arbitrary units. (B) Structures of major peaks numbered in the top chromatogram in panel A. LDT products are muropeptides containing 3-3 cross-links (peaks 4 to 7), tripeptides (peaks 1, 5, and 7) and glycine at position 4 (Gly4, peaks 2 and 4). G, *N*-acetylglucosamine; M(r), *N*-acetylmuramitol; L-Ala, L-alanine; D-Glu, D-glutamic acid; D-Ala, D-alanine; *m*-DAP, *meso*-diaminopimelic acid. The detected muropeptides with tripeptides or glycine at position 4 (peaks 2 and 4) are typical products of side reactions in PG from cells with active LDTs (due to LD-CPase and Ala-Gly exchange reactions, respectively).

We next ectopically expressed *ldtD*, *ldtE*, and/or *ldtF* (Table S1) in an *E. coli* BW25113Δ6LDT background, which lacks *ldtABCDEF* (*ldtA*-*F*) (47), and analyzed the PG composition (Fig. 2). Expression of LdtD alone, but not expression of LdtE or LdtF, resulted in the presence of 3-3 cross-links in PG. Coexpression of LdtF with LdtD or LdtE increased the level of 3-3 cross-links (compared to LdtD or LdtE alone), suggesting that LdtF might not be an active LD-TPase but stimulates the other two enzymes. In line with this hypothesis, we found that 3-3 cross-links were not detected in a Δ*ldtA-E* mutant that expressed *ldtF* as the sole *ykuD* homologue (Table S3).

10.1128/mBio.02729-18.7 TABLE S1 Bacterial strains and plasmids used in this study. Download Table S1, DOCX file, 0.03 MB. Copyright © 2019 Morè et al. 2019 Morè et al. This content is distributed under the terms of the Creative Commons Attribution 4.0 International license.

### LDTs prevent cell lysis upon defective OM assembly.

Because the level of the 3-3 cross-links increased in LptC-depleted cells, we deleted every *ldt* gene alone and all possible combinations in the *araB*p*lptC* conditional mutant, and we examined the growth profile and level of 3-3 cross-links in the PG under permissive and nonpermissive conditions.

Upon shifting to the nonpermissive condition, LptC-depleted *ldt* mutant cultures (with the exception of Δ*ldtE* Δ*ldtF* mutant) decreased in optical density, and the cells lost viability as shown by their reduced ability to form colonies. Phase-contrast and fluorescence microscopy revealed bulges at variable positions on the cell surface, suggesting that the cellular integrity was compromised (Fig. 1C and F and Fig. S2 to S4). These effects were specific for the loss of LDTs forming 3-3 cross-links because the simultaneous removal of all Lpp attachment enzymes (*ldtA-C* deletion) did not result in lysis upon *lptC* depletion (Fig. S5A and B). In the lysis-prone *lptC*-depleted Δ*ldtD* or Δ*ldtE* mutants, the level of 3-3 cross-links was only slightly reduced compared to the *araB*p*lptC* parental strain (Table 1 and Table S3). The lysis phenotype of *araB*p*lptC* Δ*ldtD* cells was rescued by ectopic expression of native LdtD, but not LdtD^C528A^ in which the catalytic Cys residue is mutated to Ala (Fig. S5C), showing that the activity of LdtD is required to rescue cells from lysis upon OM defective assembly.

10.1128/mBio.02729-18.2 FIG S2 Deletion of *ldtD*, *ldtE*, and *ldtD ldtE* in the *araB*p*lptC* conditional strain compromises cell viability under nonpermissive conditions. Download FIG S2, PDF file, 0.4 MB. Copyright © 2019 Morè et al. 2019 Morè et al. This content is distributed under the terms of the Creative Commons Attribution 4.0 International license.

10.1128/mBio.02729-18.3 FIG S3 Phenotypes of wild-type BW25113 (*lptC*^+^) and *araB*p*lptC* conditional strains lacking *ldtF* or *ldtD ldtF*. Download FIG S3, PDF file, 0.4 MB. Copyright © 2019 Morè et al. 2019 Morè et al. This content is distributed under the terms of the Creative Commons Attribution 4.0 International license.

10.1128/mBio.02729-18.4 FIG S4 Growth profiles and cell imaging of *araB*p*lptC* Δ*ldtE* Δ*ldtF* and *araB*p*lptC* Δ*lpoB* strains and growth profile of the *araB*p*lptC* Δ*cpoB* strain. Download FIG S4, PDF file, 0.3 MB. Copyright © 2019 Morè et al. 2019 Morè et al. This content is distributed under the terms of the Creative Commons Attribution 4.0 International license.

10.1128/mBio.02729-18.5 FIG S5 Growth profiles of *araB*p*lptC* Δ*ldtA* Δ*ldtB* Δ*ldtC* and *araB*p*lptC* Δ*ldtA* Δ*ldtB* Δ*ldtC* Δ*ldtD* Δ*ldtE* Δ*ldtF* strains. The *araB*p*lptC* strain with different combinations of *ldt* genes deleted was complemented by ectopic expression of wild-type *ldtD*. Download FIG S5, PDF file, 0.4 MB. Copyright © 2019 Morè et al. 2019 Morè et al. This content is distributed under the terms of the Creative Commons Attribution 4.0 International license.

All strains with defective *ldt* genes lysed under nonpermissive conditions except the *araB*p*lptC* Δ*ldtE* Δ*ldtF* mutant which arrested growth like the *araB*p*lptC* parental strain (Fig. 1F and Fig. S4A and B). These cells displayed a high level (>8%) of 3-3 cross-links at all conditions (i.e., even without depletion of *lptC*) (Table 1). This suggests that LdtD is active and able to prevent lysis of these cells. In line with this finding, we indeed observed that ectopic expression of *ldtD* rescues all LptC-depleted single and multiple *ldt* mutant strains from lysis (Table 1 and Fig. S5D to H). Finally, the LptC-depleted Δ*ldtF* mutant produced 3-3 cross-links and lysed at nonpermissive conditions (Table 1 and Fig. S3C and D), but in sharp contrast to the other strains, *araB*p*lptC* Δ*ldtF* cells showed morphological defects even when grown at permissive conditions (Fig. S3D), and no morphological defects were observed when *ldtF* was deleted in the *lptC*^+^ background (Fig. S3B), suggesting that the deletion of *ldtF* caused additional problems to cells with depleted LptC levels.

**FIG 3:**
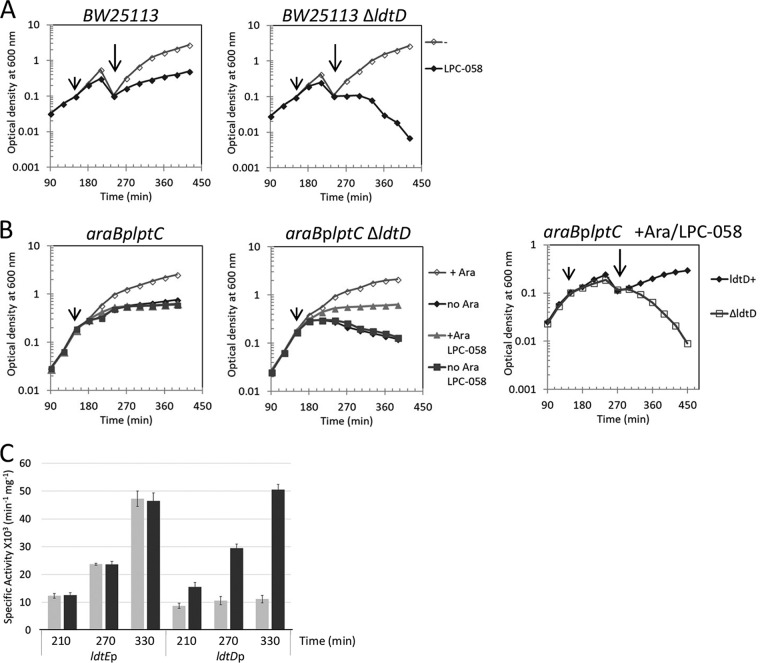
Inhibition of LPS synthesis causes lysis in *ldtD* deleted cells and activates the *ldtD* promoter. (A) *E*. *coli* BW25113 (left) and BW25113Δ*ldtD* (right) cells were grown in LB-Lennox medium. At *t* = 150 min, cells were treated with 0.031 µg/ml (1×MIC) of LPC-058 (short arrow) or not treated with LPC-058. Cell growth was monitored by OD_600_ measurements. When cells reached late exponential phase, cultures were diluted to an OD_600_ of 0.1 (long arrow), and growth was monitored by OD_600_ measurements. (B) Cells of *araB*p*lptC* (left panel) and *araB*p*lptC* Δ*ldtD* (middle and right panels) were grown in the presence of 0.2% arabinose to an OD_600_ of 0.2, harvested, washed three times, and resuspended in an arabinose-supplemented (+ Ara) or arabinose-free (no Ara) medium. Cell growth was then monitored by OD_600_ measurements. At *t* = 150 min, cells were treated with 0.006 µg/ml (0.75×MIC) of LPC-058 (short arrow) or not treated with LPC-057, and afterwards growth was monitored by OD_600_ measurements. When *araB*p*lptC* and *araB*p*lptC* Δ*ldtD* cells grown in the presence of arabinose and treated with LPC-058 (right panel) reached late exponential phase, the cultures were diluted to an OD_600_ of 0.1 (long arrow), and growth was monitored by OD_600_ measurements. Growth curves shown are representative of at least three independent experiments. (C) BW25113 cells carrying plasmids expressing *ldtD*p-*lacZ* and *ldtE*p-*lacZ* fusions were grown in LB Lennox broth. At *t* = 150 min cells were treated with 0.031 µg/ml (1×MIC) LPC-058 or not treated. β-Galactosidase specific activity was determined from cells collected at 210 min (OD_600_ of 0.5), 270 min (60 min after dilution), and 330 min (120 min after dilution). Light gray bars show strain BW25113, and gray bars show strain BW25113 treated with LPC-058. Note that *ldtE* expression is not affected by LPC-058.

Lysis of LptC-depleted cells could be caused by the accumulation of LPS at the outer leaflet of the CM (11, 14). We therefore assessed whether the LptC depletion-induced lysis occurs in cells with blocked LPS synthesis due to inhibition of LpxC by LPC-058 (48). We observed lysis in BW25113 Δ*ldtD* and *araB*pl*ptC* Δ*ldtD* cells treated with LPC-058 but not in the corresponding parental strains (carrying a functional *ldtD* copy) treated with LPC-058 (Fig. 3A and B). These results suggest that lysis is not due to perturbation of the CM or periplasmic stress caused by depletion of a component of the Lpt machinery but is rather the consequence of lack of PG remodeling by LdtD.

### The *ldtD* promoter is activated under envelope stress conditions.

To assess how the *ldt* genes are regulated in the cell, we constructed transcriptional fusions of the promoter region of each *ldt* gene to *lacZ*, and the resulting plasmids with p*ldtD*-*lacZ*, p*ldtE*-*lacZ*, and p*ldtF*-*lacZ* were introduced into strain BW25113, the conditional *araB*p*lptC* mutant, and their derivatives with deletion of *ldtD*, *ldtE*, and *ldtF* alone and in all possible combinations. β-Galactosidase activity was measured in extracts from cells collected at different time points during growth.

**FIG 4:**
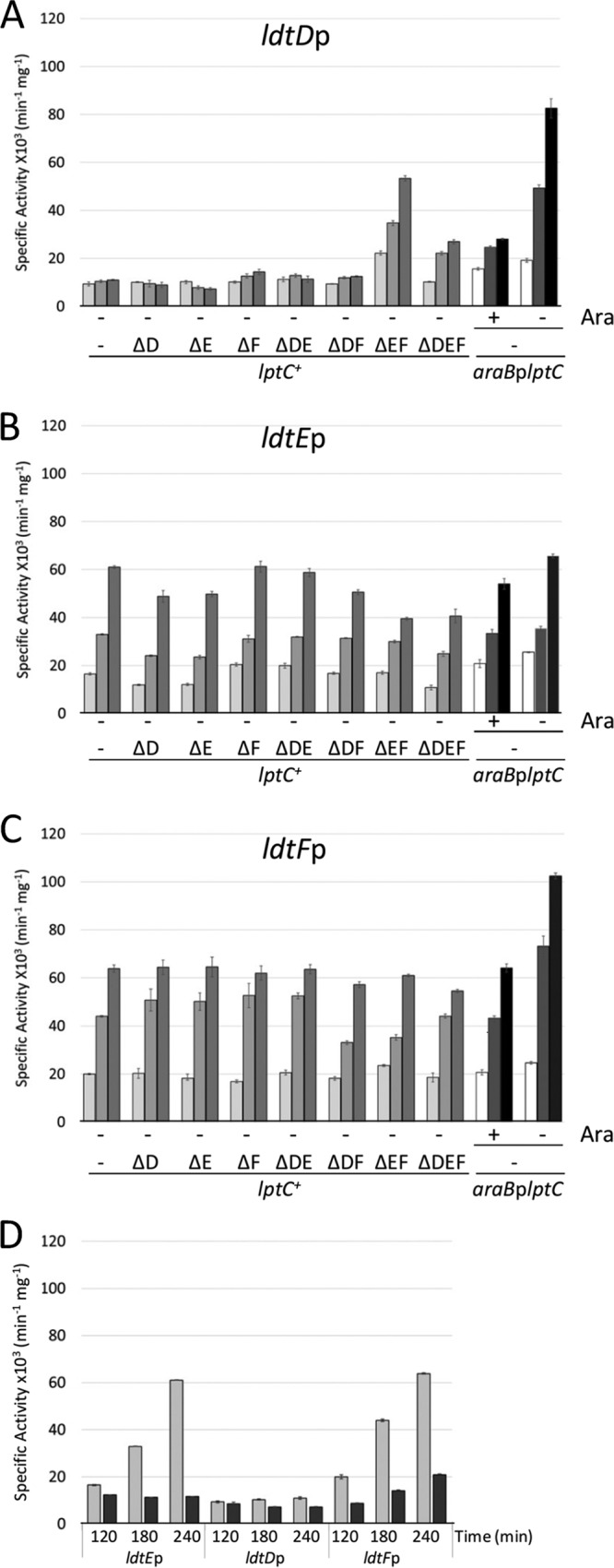
The *ldtD* promoter is activated under envelope stress conditions, and *ldtE* and *ldtF* are RpoS-regulated genes. Wild-type strain BW25113 (*lptC*^+^) and isogenic mutants with every *ldt* gene deleted alone and in all possible combinations were transformed with plasmids expressing *ldtD*p-*lacZ* (A), *ldtE*p-*lacZ* (B), or *ldtF*p-*lacZ* (C) fusions. Cells were grown in LD medium. β-Galactosidase specific activity was calculated from cells collected at 120 min (OD_600_ of ∼0.2) (light gray bars), 180 min (OD_600_ of ∼0.8) (gray bars), and 210 min (OD_600_ of ∼2.0) (dark gray bars) (left side). The *araB*p*lptC* conditional strain was transformed with plasmids expressing *ldtD*p-*lacZ* (A), *ldtE*p-*lacZ* (B), or *ldtF*p-*lacZ* (C). Cells were grown with 0.2% arabinose to an OD_600_ of 0.2, harvested, washed three times, and resuspended in an arabinose-supplemented (+ Ara) or arabinose-free (− Ara) medium. Samples for determination of β-galactosidase specific activity were collected at the time point at which the strains cultivated under nonpermissive conditions arrested growth (white bars) and 30 min (gray bars) and 60 min (black bars) afterwards (+Ara and no Ara conditions, right side). (D) BW25113 Δ*rpoS* cells carrying plasmids expressing *ldtD*p-*lacZ*, *ldtE*p-*lacZ*, or *ldtF*p-*lacZ* fusions were grown in LD broth. β-Galactosidase specific activity was determined from cells collected at 120 min (OD_600_ of 0.2), 180 min (OD_600_ of 0.8), and 210 min (OD_600_ of 2.0). Strains BW25113 (light gray bars) and BW25113Δ*rpoS* (gray bars) are shown. Note that *ldtD* expression is not affected in a Δ*rpoS* background. The values are the means ± SD from at least three independent experiments. All mutants were also transformed with the empty plasmid, and the mean of β-galactosidase specific activity calculated from cells grown in any condition was 249 ± 30 (min^−1 ^mg^−1^). In panels A to C, the *ldt* genes are indicated by their loci shown in capital letters.

The expression of *ldtE* and *ldtF* in the *lptC*^+^ background was growth phase dependent. In the wild-type strain and the *araB*p*lptC* conditional mutant grown under permissive and nonpermissive conditions, p*ldtE*-*lacZ* and p*ldtF*-*lacZ* were maximally expressed in stationary-phase cells (Fig. 4B and C). Consistent with their expression pattern, *ldtE* and *ldtF* were both regulated by RpoS, the alternative sigma factor for stationary-phase gene expression (49), and both genes lost their growth phase-dependent promoter activation in a BW25113Δ*rpoS* mutant (Fig. 4D).

The *ldtD* promoter was not activated in the wild-type *lptC*^+^ strain and in *ldt* derivatives with the exception of the Δ*ldtE* Δ*ldtF* mutant and was up to eightfold activated by LptC depletion (Fig. 4A). We also observed *ldtD* but not *ldtE* activation in wild-type BW25113 cells carrying p*ldtD*-*lacZ* or p*ldtE*-*lacZ* and treated with LPC-058 (Fig. 3C).

In summary, *ldtE* and *ldtF* are housekeeping LDTs which share a growth phase-dependent activation profile under all conditions tested, and their expression was unaffected by the presence or absence of arabinose in the *araB*p*lptC* conditional strain. In contrast, *ldtD* was strongly expressed in the *lptC*^+^ background in which both *ldtE* and *ldtF* were deleted, in LptC-depleted cells, and in cells with blocked LPS synthesis. Hence, LdtD is the stress LDT activated under cell envelope stress conditions or in the absence of the housekeeping LdtE/LtdF, consistent with the presence of increased levels of 3-3 cross-links under these conditions.

### Growth arrest without lysis requires PG synthesis and maturation.

Thus far, our data suggest that LDTs play a major role in PG remodeling in protecting cells from lysis upon LPS export pathway defects. LDTs can facilitate PG growth in certain β-lactam-resistant strains of E. coli and E. faecium, and in this situation, they function with a GTase domain of a bifunctional PG synthase, and a DD-CPase (42–45). LptC-depleted cells have been shown previously to have elevated levels of the bifunctional PBP1B and the DD-CPases PBP5 and PBP6a (46). PBP5 is the major DD-CPase active under standard laboratory conditions (32). PBP6a is an additional DD-CPase with an unknown physiological function, as it does not seem to be active under standard growth conditions (35).

**FIG 5:**
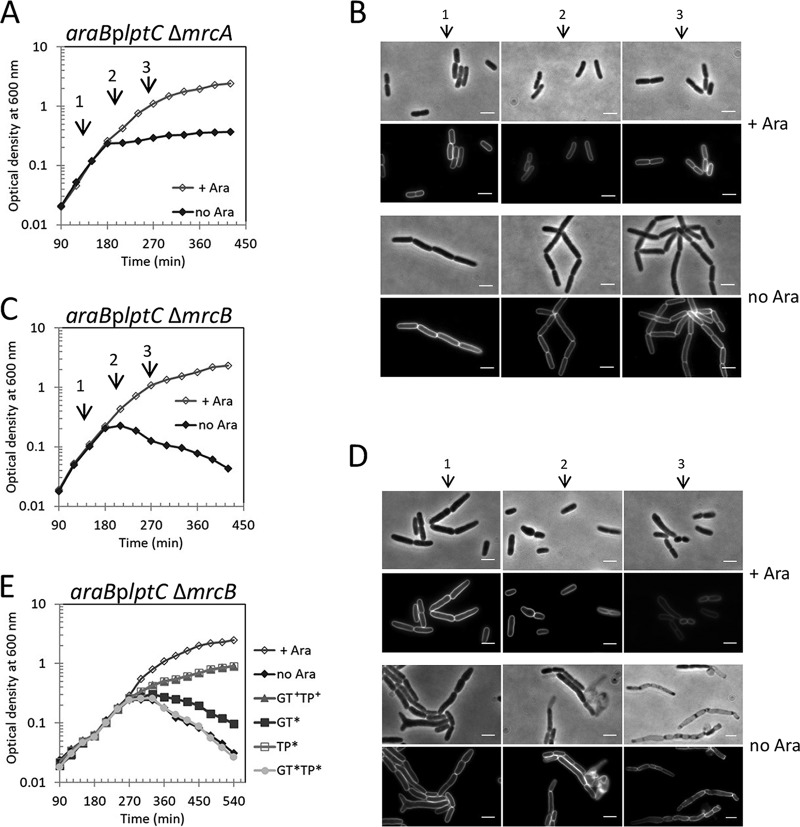
The GTase activity of PBP1B is required to prevent cell lysis upon defective OM assembly. Cultures of *araB*p*lptC* Δ*mrcA* (A) or *araB*p*lptC* Δ*mrcB* (C) strains lacking PBP1A and PBP1B, respectively, were grown with 0.2% arabinose to an OD_600_ of 0.2, harvested, washed three times, and resuspended in an arabinose-supplemented (+ Ara) or arabinose-free (no Ara) medium. Cell growth was then monitored by OD_600_ measurements. At *t* = 120 min, 210 min, and 270 min (arrows 1, 2, and 3, respectively), samples from *araB*p*lptC* Δ*mrc*A (B) and *araB*p*lptC* Δ*mrc*B (D) strains were collected for imaging. Phase-contrast images (top) and fluorescence images (bottom) are shown. Bars, 3 μm. (E) Complementation of the *araB*p*lptC* Δ*mrcB* lysis phenotype by ectopic expression of wild-type *mrcB* (GT^+^TP^+^), *mrcB* with mutated GTase (GT*), TPase (TP*), or both (TP*GT*). All mutants were grown in the presence of 0.2% arabinose at 30°C to an OD_600_ of 0.2, harvested, washed three times, and resuspended in an arabinose-free medium. The growth of the *araB*p*lptC* Δ*mrcB* strain in arabinose-supplemented medium is shown as a control. Cell growth was monitored by OD_600_ measurements. Growth curves shown are representative of at least three independent experiments.

We next asked whether bifunctional PBPs and DD-CPases are important to prevent lysis in LptC-depleted cells, as are the LDTs. PBP1B, but not PBP1A, was required to prevent lysis of LptC-depleted cells (Fig. 5A to D), and lysis could be prevented by ectopic expression of PBP1B (Fig. 5E). We next tested which of the two activities of PBP1B was needed to prevent lysis. The ectopic expression of PBP1B(S510A) with an active GTase and inactive TPase domain was fully functional in preventing lysis, showing that the TPase activity of PBP1B is not required (Fig. 5E). However, the ectopic expression of PBP1B(E233D) with inactive GTase function was unable to prevent lysis of LptC-depleted cells lacking wild-type PBP1B, suggesting that the GTase activity of PBP1B is crucial to prevent lysis (Fig. 5E). Consistent with this conclusion, lysis was also observed in cells lacking LpoB, a key activator of the GTase of PBP1B (26, 28, 50) (Fig. S4C and D). Another regulator of PBP1B, CpoB (27), was not required to prevent lysis upon LptC depletion (Fig. S4E), consistent with CpoB’s exclusive regulation of the TPase function of PBP1B and our findings that TPase was not needed to prevent lysis.

**FIG 6:**
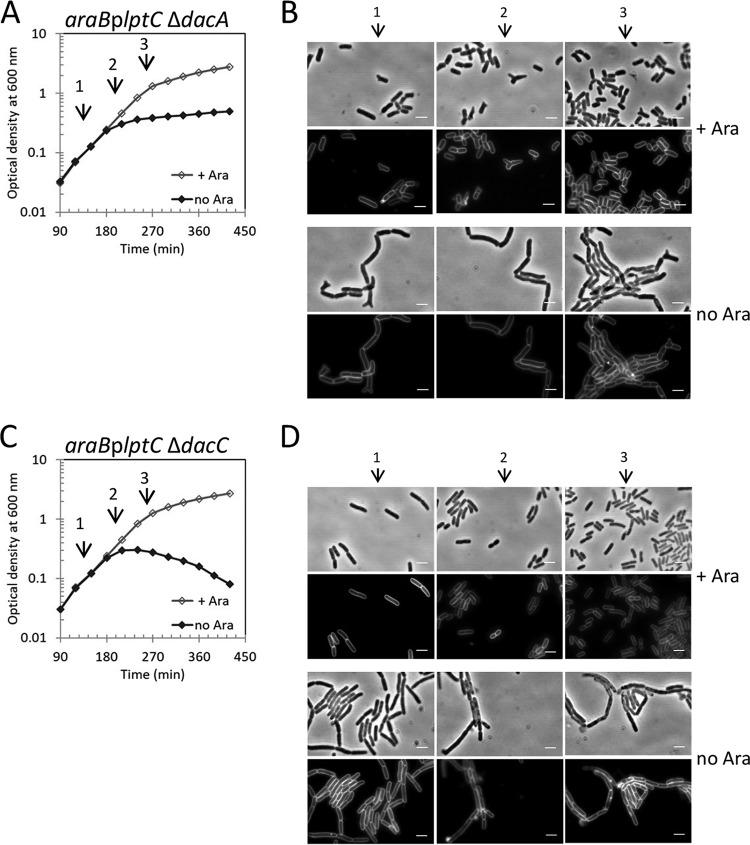
The DD-CPase PBP6a prevents cell lysis upon defective OM assembly. Cells of the *araB*p*lptC* Δ*dacA* (A) or *araB*p*lptC* Δ*dacC* (C) strain lacking PBP5 or PBP6a, respectively, were grown in the presence of 0.2% arabinose to an OD_600_ of 0.2, harvested, washed three times, and resuspended in an arabinose-supplemented (+ Ara) or arabinose-free (no Ara) medium. Cell growth was then monitored by OD_600_ measurements. Growth curves shown are representative of at least three independent experiments. At *t* = 120 min, 210 min, and 270 min (arrows 1, 2, and 3, respectively), samples from *araB*p*lptC* Δ*dacA* (B) and *araB*p*lptC* Δ*dacC* (D) strains were collected for imaging. Phase-contrast images (top) and fluorescence images (bottom) are shown. Bars, 3 μm.

Finally, survival of LptC-depleted cells required the DD-CPase gene *dacC*, encoding PBP6a, but not the *dacA* gene encoding PBP5 (Fig. 6). Therefore, preventing lysis upon severe LPS transport defect requires not only LDTs but also the GTase activity of PBP1B and the DD-CPase PBP6a, presumably to synthesize and to modify the nascent PG substrate for the LDTs.

### LdtD interacts with PBP1B.

**FIG 7:**
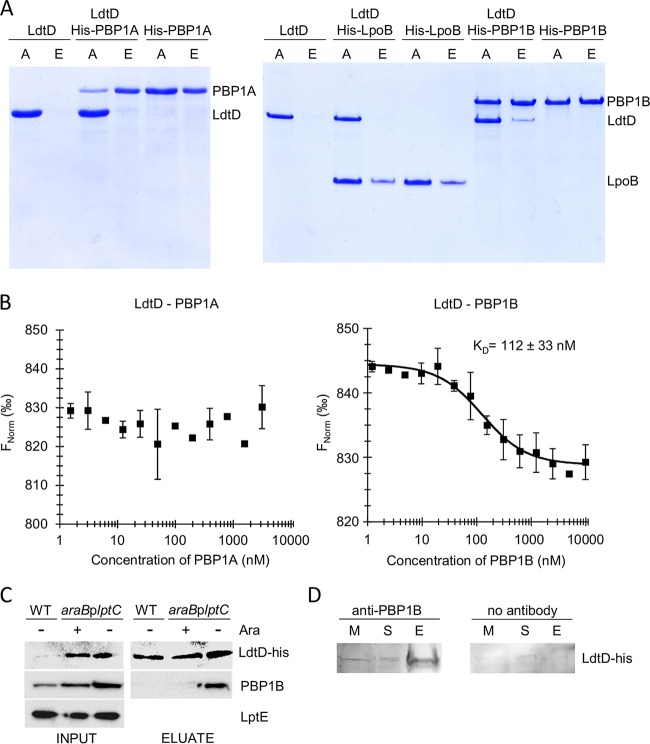
LdtD interacts with PBP1B *in vitro* and *in vivo*. (A) Coomassie blue-stained SDS-PAGE gel showing the pulldown of proteins to Ni^2+^-NTA beads. LdtD bound to the beads and was present in the elution fraction (lanes E) only in the presence of oligohistidine-tagged PBP1B, and not in the presence of oligohistidine-tagged LpoB, oligohistidine-tagged PBP1A, or in the absence of another protein. A, applied sample. (B) Microscale thermophoresis curves showing that LdtD interacts with PBP1B and not with PBP1A. The *K_D_* value for the LdtD-PBP1B interaction is indicated. Values are means ± SD from three independent experiments. (C). BW25113 *ldtD*-*his* and *araB*p*lptC ldtD*-*his* cells grown with and without arabinose (Ara) were treated with the DTSSP cross-linker. Cell-free extract was prepared, and LdtD-His was purified onto a Ni-NTA resin. PBP1B, LdtD-His, and LptE (as loading control) were immunodetected after SDS-PAGE and Western blotting. WT, wild type. (D) *In vivo* interaction between PBP1B and LdtD-His by cross-linking/coimmunoprecipitation assay. *araB*p*lptC ldtD-his* cells were treated with cross-linker DTSSP. The membrane fraction was prepared, and PBP1B was precipitated by specific antibody (the control sample received no antibody). LdtD-His was detected by Western blotting using specific anti-oligohistidine-tag antibody. M, membrane extract; S, supernatant; E, elution.

Our data supported the hypothesis that LdtD may function with PBP1B to rescue sacculus integrity upon severe OM assembly defects. We then asked whether LdtD physically interacts with class A PBPs by mixing purified oligohistidine-tagged PBP1A or PBP1B with untagged LdtD and assaying binding to Ni^2+^-NTA beads. LdtD was pulled down by oligohistidine-tagged PBP1B, not by oligohistidine-tagged PBP1A or LpoB, or in the absence of tagged protein (Fig. 7A), suggesting a direct interaction with oligohistidine-tagged PBP1B. The pulldown was confirmed and extended by microscale thermophoresis, which revealed an interaction between LdtD and PBP1B, but not between LdtD and PBP1A. The *K_D_* value of the LdtD-PBP1B interaction was 112 ± 33 nM (Fig. 7B). Moreover, PBP1B was pulled down by oligohistidine-tagged LdtD, expressed from the chromosome from its native promoter, only upon LptC depletion (Fig. 7C), and LdtD and PBP1B interacted in LptC-depleted cells as shown by chemical cross-linking followed by immunoprecipitation (Fig. 7D). These data suggest that a PBP1B-LdtD complex is formed in cells experiencing an OM assembly defect.

### LdtD forms 3-3 cross-links in mature and nascent PG.

**FIG 8:**
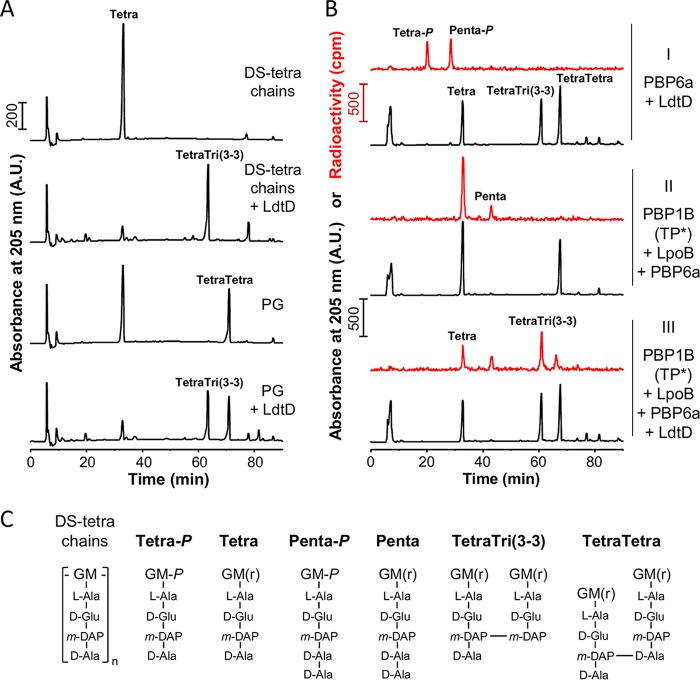
LdtD shows LD-TPase activity with different PG substrates. (A) HPLC chromatograms showing the formation of TetraTri(3–3) dimers by LdtD incubated with glycan chains harboring monomeric tetrapeptides (DS-tetra chains) or PG from BW25113Δ6LDT cells lacking all six *ldt* genes. Samples were digested with cellosyl and, reduced with sodium borohydride before HPLC analysis. (B) HPLC chromatograms obtained from samples upon incubating [^14^C]Glc*N*Ac-labeled lipid II and PG from strain BW25113Δ6LDT and the proteins indicated to the right (I, II, and III indicate the different samples). Samples were digested with cellosyl, reduced with sodium borohydride, and subjected to HPLC analysis with detection of both UV signal (black traces) and radioactivity (red traces). PBP1B (TP*) is PBP1B with an inactive transpeptidase site due to the replacement of Ser-510 by Ala. Tetra-*P* and Penta-*P* originate from the hydrolysis of the respective pentapeptide and tetrapeptide versions of lipid II prior to HPLC analysis. (C) Proposed structures of muropeptides present in the fractions in panels A and B. G, *N*-acetylglucosamine; M, *N*-acetylmuramic acid; M(r), *N*-acetylmuramitol; M-*P*, N-acetylmuramic acid-1-phosphate; L-Ala, L-alanine; D-Glu, D-glutamic acid; D-Ala, D-alanine; *m*-DAP, *meso*-diaminopimelic acid.

The LDT activity of LdtD has been demonstrated previously with a soluble disaccharide tetrapeptide substrate (45). Considering its role in PG remodeling and its interaction with PBP1B, we hypothesized that the enzyme must be active against larger PG fragments or even sacculi and/or nascent PG produced by PBP1B. We tested these possibilities by first incubating LdtD with either soluble glycan chains carrying non-cross-linked tetrapeptides (DS-tetra chains, the products of MepM [Fig. 8A]) and PG sacculi purified from strain BW25113Δ6LDT. LdtD was highly active against these substrates (Fig. 8A), utilizing almost all monomeric tetrapeptides to generate the 3-3 cross-linked dimer (disaccharide tetratripeptide, TetraTri). The high activity is particularly remarkable in the case of the sacculi, which after the reaction with LdtD contained an unusually high cross-linkage with ∼84% of all muropeptides present in cross-links.

We next assayed the activity of LdtD during synthesis of PG *in vitro* using radiolabeled lipid II as the substrate in the presence of 10-fold excess of unlabeled PG sacculi. After the reaction, the products were digested with the muramidase cellosyl, and the resulting muropeptides were separated by HPLC using back-to-back UV and radioactivity detectors to monitor the products formed. LdtD produced a highly 3-3 cross-linked nascent PG, as seen by the abundant radiolabeled TetraTri(3-3) muropeptide present in the reaction with the TPase-inactive PBP1B(S510A) mutant, its activator LpoB, and the DD-CPase PBP6a (red trace in sample III [Fig. 8B]). In the absence of LdtD, PBP1B(S510A)/LpoB produced non-cross-linked glycan chains with pentapeptides of which most were trimmed by PBP6a to tetrapeptides (red traces in sample II [Fig. 8B]), and no 3-3 cross-links were observed in the UV traces (sample II [Fig. 8B]). Remarkably, LdtD preferentially acted on the nascent (radioactive) PG (red trace, sample III) despite the presence of an ∼10-fold excess of unlabeled PG sacculi (black trace, sample III). The UV traces showed that ∼52% of the unlabeled tetrapeptides were consumed by LdtD (comparing the black traces in samples II and III [Fig. 8B]), which was markedly less than the ∼68% consumption of the radiolabeled tetrapeptides (comparing the red traces in samples II and III [Fig. 8B]). This suggests that LdtD prefers new PG, synthesized by PBP1B and trimmed by PBP6a, as the substrate. LdtD showed similar activity in reactions with PBP5 (instead of PBP6a), showing that both DD-CPases are capable of providing the tetrapeptide substrates (Fig. S6).

10.1128/mBio.02729-18.6 FIG S6 LdtD is active during *in vitro* PG synthesis in the presence of PBP1B(TP*), LpoB, and PBP5. Download FIG S6, PDF file, 0.2 MB. Copyright © 2019 Morè et al. 2019 Morè et al. This content is distributed under the terms of the Creative Commons Attribution 4.0 International license.

Together, the results of the activity assays support the phenotypic data and muropeptide analysis showing that LdtD is highly active in producing 3-3 cross-links in PG sacculi, and it is able to cooperate with the GTase activity of PBP1B and DD-CPases to utilize nascent PG as the substrate, consistent with a role in protective remodeling of PG during OM defective assembly.

## DISCUSSION

LPS is essential in many Gram-negative bacteria with several notable exceptions, namely Neisseria meningitidis (51), Moraxella catarrhalis (52) and Acinetobacter baumannii (53), which can grow without LPS. E. coli requires LPS, and therefore, the depletion of LptC is not compatible with cell growth. However, although cells are unable to continue growing, they do survive the block of LPS transport for several hours, and they resume growth once expression of LptC is restored.

In this work, we discovered a PG remodeling pathway involving LDTs that is essential for survival in cells with defective OM assembly, revealing a link between LPS export and a dedicated mode of PG synthesis. LDTs are not required in unstressed cells which, however, do remodel the PG to introduce a small number of 3-3 cross-links upon entry into stationary phase, perhaps to repair minor defects in PG. Expanding from previous work (39–41), we also show here that E. coli has an additional YkuD homologue, LdtF, which is not an active LD-TPase *per se* but might stimulate other LDTs.

### Roles of the different LDTs.

LdtE is the housekeeping LDT that is induced by RpoS when cells enter stationary phase (Fig. 4) consistent with the increase in 3-3 cross-links in stationary-phase cells (36, 54). LdtE seems to require LdtF for activity (Fig. 2) and the LdtE-LdtF couple forms most of the 3-3 cross-links in unstressed cells in which LdtD is poorly expressed (Table 1 and Fig. 4).

LDTs become essential to prevent cell lysis in LptC-depleted cells which upregulate *ldtD* and increase 3-3 cross-links (Fig. 4 and Table 1). Notably, LDTs are inhibited by sub-MIC copper ions which therefore reduce the robustness of the cell envelope to withstand LPS export stress (55). That LdtD plays a major role in PG remodeling during cell envelope stress is consistent with its induction by the Cpx-mediated stress response (56, 57). The single Δ*ldtE* or Δ*ldtF* mutants lysed upon LptC depletion despite the presence of a functional copy of *ldtD*; presumably, they are unable to accumulate sufficient LdtD activity to avoid lysis upon LptC depletion. In contrast, the Δ*ldtE* Δ*ldtF* double mutant is already stressed and has a high level of LdtD (and of 3-3 cross-links) before the depletion of LptC, preventing lysis once LptC is depleted (Table 1). This conclusion is further supported by the finding that ectopic expression of *ldtD* prevents lysis of all single and multiple *ldt* mutants depleted for LptC. For unknown reason, the *ldtF* mutant shows impaired cell morphology even before LptC depletion (see Fig. S3D in the supplemental material), suggesting that enhanced 3-3 cross-links are not always protective and that LdtF, which has been implicated in biofilm formation in enteroaggregative E. coli (58), has an additional role in the cell. Hence, our PG analysis highlights that an increased level of 3-3 cross-links cannot protect every *ldt* mutant cell from lysis, but importantly, the ability of cells to avoid lysis is always accompanied by an increase in 3-3 cross-links (Table 1).

### LdtD is part of a “PG repair machine” with PBP1B/LpoB and PBP6a.

**FIG 9:**
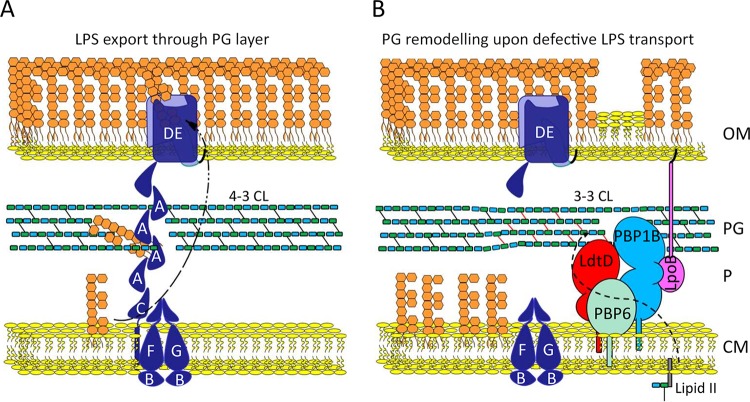
Role of a PG repair machine. (A) Nonperturbed LPS transport to the OM. (B) Upon LptC depletion, PBP1B-LpoB, LdtD, and PBP6a work in concert to repair the PG, synthesizing it locally with 3-3 cross-links (CL) (red line). Components of the Lpt machine are colored blue and indicated by capital letters.

LptC-depleted cells also required the GTase function of PBP1B, its activator LpoB, and the DD-CPase PBP6a (but not PBP1A or PBP5) to avoid lysis (Fig. 5 and 6). To our knowledge, this is the first condition where PBP6a becomes important. Our genetic evidence (Fig. 5C and D), the previously observed induction of the PBP1B and PBP6a genes in LptC-depleted cells (46), and the physical interaction of LdtD with PBP1B *in vitro* and in stressed cells (Fig. 7) all support a model in which PG remodeling machinery containing PBP1B/LpoB, LdtD, and PBP6a polymerizes PG strands (GTase of PBP1B), trims the pentapeptides (PBP6a), and utilizes the resulting tetrapeptides to form 3-3 cross-links (LdtD) (Fig. 9).

### How does PG remodeling rescue cells from lysis?

The PG layer is an elastic, net-like structure thought to be the major stress-bearing structure in the bacterial cell envelope allowing the cell to sustain large mechanical loads such as turgor pressure (37, 59). This prevailing dogma has been challenged by studies of phage lysis (60, 61) and more recently by Rojas and coworkers who showed that the OM and PG balance the mechanical loads during osmolality changes (62). Interestingly, a mutant defective in LPS export carrying the *imp4213* allele of *lptD* (63) produced an OM with an altered load-bearing capacity (62). Defects in the OM (i.e., perturbation of LPS layer and local loss of lipid asymmetry) may cause local mechanical stress on the PG structure, and hence, the LDT-mediated PG remodeling could strengthen the PG to rebalance the mechanical load between the OM and cell wall.

We also envision another possible reason why LDTs are essential upon defective LPS export. The size of pores in the PG net is too small for large transenvelope assemblies, such as the flagella and type II secretion systems, and hence, the assembly of these requires the local hydrolysis of the PG to increase the pore size (64, 65). The width of the periplasmic Lpt “bridge” together with its bulky LPS cargo (19, 66) is likely wider than the diameter of pores in PG (4.1 to 6.2 nm, depending on the turgor), necessitating the local hydrolysis of the PG, by an as yet unknown PG hydrolase, for the assembly of the Lpt machinery and rapid flux of LPS to the cell surface. The Lpt complex is known to disassemble when LPS transport is arrested due to depletion of LptC (14, 17, 18). Hence, it is possible that LDTs seal (repair) the PG locally after the disassembly of LPS export machines. We propose a dedicated PG repair machine, containing PBP1B/LpoB, LdtD, and PBP6a for this function (Fig. 9). The sequence of events that lead to cell lysis following the block of LPS biogenesis in the absence of LDTs are currently not known, but our data suggest that lysis is likely the consequence of the accumulation of defects in the PG that cannot be repaired and lead to unbalanced mechanical load between the OM and PG.

The GTase function of the PG repair machine is activated by the OM-anchored lipoprotein LpoB, which spans the periplasm to interact with the UB2H domain of PBP1B. Hence, apart from its role in the synthesis of “normal PG” (with 4-3 cross-links) during cell elongation and division, the PBP1B-LpoB system has another role in PG remodeling together with LdtD, producing PG with 3-3 cross-links. PBP1B/LpoB, LdtD, and the DD-CPase PBP5 enabled an E. coli mutant strain to grow in the presence of an otherwise lethal concentration of ampicillin (45), and PBP1B/LpoB (and not PBP1A/LpoA) promoted the recovery of PG-less L-form cells of E. coli to the walled state, generating a PG layer *de novo* (67). These observations and our own work highlight the versatility of the PBP1B/LpoB PG synthase/regulator pair, which is used by the cell in different processes and circumstances. PBP1A/LpoA are able to compensate for the loss of PBP1B/LpoB in normal growth, but they cannot compensate for the stress-related function of PBP1B/LpoB with LptD. Indeed, cells in which PG synthesis may be considered “weakened” by the lack of PBP1A or PBP5 do survive LPS transport defects just as well as wild-type cells. Hence, our combined data support a specific PG remodeling mechanism instead of nonspecific effects such as a general “weakening” of PG synthesis.

In summary, we discovered a role of 3-3 cross-links in the PG as a mean to fortify the sacculus in response to severe OM assembly defects. This functional connection between OM biogenesis and PG remodeling highlights an elegant and versatile mechanism bacteria employ to maintain the integrity of their essential cell envelope under a variety of growth and stress conditions.

## MATERIALS AND METHODS

### Bacterial strains, plasmids, and growth conditions.

Bacterial strains and plasmids used in this work are listed in Table S1 in the supplemental material. Primers used are listed in Table S2. Cells were routinely grown aerobically at 37°C or 30°C in LB-Lennox medium (10 g/liter tryptone, 5 g/liter yeast extract, 5 g/liter NaCl) (Difco). When required, antibiotics or inducers were added: ampicillin (100 μg/ml), chloramphenicol (25 μg/ml), kanamycin (25 μg/ml), arabinose (0.2% [wt/vol]), IPTG (0.1 mM). For LptC depletion, bacteria were harvested from cultures with an OD_600_ of 0.2 by centrifugation, washed twice with LD, and diluted 100-fold in LD with or without arabinose. Cell growth was monitored by OD_600_ measurements, and viability was determined by quantifying the colony-forming units (CFU).

10.1128/mBio.02729-18.8 TABLE S2 Oligonucleotides used in this study. Download Table S2, DOCX file, 0.01 MB. Copyright © 2019 Morè et al. 2019 Morè et al. This content is distributed under the terms of the Creative Commons Attribution 4.0 International license.

10.1128/mBio.02729-18.9 TABLE S3 Muropeptide composition of *ldt* mutant strains with or without (separate file) depletion of *lptC*. Download Table S3, XLSX file, 0.03 MB. Copyright © 2019 Morè et al. 2019 Morè et al. This content is distributed under the terms of the Creative Commons Attribution 4.0 International license.

The phenotypes of *araB*p*lptC* and isogenic *ldt*s mutant derivatives were summarized as the slope of each growth curve between 180 and 390 minutes (Fig. 1F). Each slope was calculated as the regression line based on the data points identified by *y* values (expressed as absorbance at 600 nm) and *x* values (time expressed in hours) using Excel functions.

### Other methods.

The construction of plasmids and strains, microscopy of cells, protein purification and biochemical assays are described in detail in Text S1 in the supplemental material.

10.1128/mBio.02729-18.10 TEXT S1 Details of the methods of strain and plasmid construction, protein purification procedures, protein-protein interaction protocols, and activity assays. Download Text S1, PDF file, 0.5 MB. Copyright © 2019 Morè et al. 2019 Morè et al. This content is distributed under the terms of the Creative Commons Attribution 4.0 International license.
