# Supplementary material for: Peptidoglycan Remodeling Enables Escherichia coli To Survive Severe Outer Membrane Assembly Defect
Source: mBio. 2019 Feb 5;10(1):e02729-18. doi: 10.1128/mBio.02729-18 (PMC6428754; doi:10.1128/mBio.02729-18)
Supplement: FIG S1 [file mBio.02729-18-sf001.pdf]

A

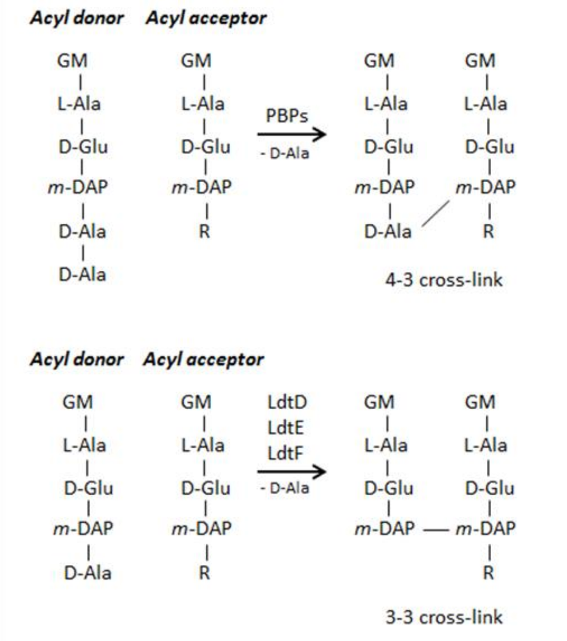

B

```
LdtD      GQLPTFVAGLAPQHPQYAAMHESLLALLCDTKPWPQLTGKATLRPGQWSNDVPALREILQ
LdtE      -------------------MKRASLL-T------LTLIGAFSAIQAAWAVDYPLPPTG--
LdtF      -------------------MRKIALILA------MLLIPCVSF-----------------
                             *:.  *           *    :

LdtD      RTGMLDGGPKITLPGDDTPTDAVVSPSAVTVETAETKPMDKQTTSRSKPAPAVRAAYDNE
LdtE      -SRLVGQNQTY------------------TVQ-----------------------EGDKN
LdtF      -AGL--------------------------------------------------------
           : :

LdtD      LVEAVKRFQ--AWQGLGADGAIGPATRDWLNVTPAQRAGVLALNIQRLRLLPTELSTGIM
LdtE      LQAIARRFDTAAMLILEANNTIAPVPKPGT-----------TITIPSQLLLPDAPRQGII
LdtF      ---------------LGSSSSTTPVSKEYK-----------Q-----Q-LMGSPVYIQIF
                         * :..:  *. :                      *:       *:

LdtD      VNIPAYSLVYYQNGNQ--VLDS-RVIVGRPDRKTPMMSSALNNVVVNPPWNVPPTLAR--
LdtE      VNLAELRLYYYPPGEN--IVQVYPIGIGLQGLETPVMETRVGQKIPNPTWTPTAGIR---
LdtF      KEERTLDLY-VKMGEQYQLLDSYKICKYSGG---------LGPKQRQGDFKSPEGFYSVQ
           :     *     *::  :::   :     .         :.    :  :.    :

LdtD      -KDILPKVRNDPGYLESHGYTVMRGWNSREAIDPWQVDWSTITASNLPFRFQQAPGPRNS
LdtE      QRSLERG---------------------------------------IKLPPVVPAGPNNP
LdtF      RNQLKPD---------------------------------------SRYYKAINIGFPNA
           ..:                                                   *  *

LdtD      LGRYKFNMPSS-EAIYLHDTPNHNLFKRDTRALSSGCVRVNKASDLANMLLQDAGWNDKR
LdtE      LGRYALRLAHG-NGEYLIHGTS--APDSVGLRVSSGCIRMNAPDIKALFSSVRTGTPVKV
LdtF      -----YDRAHGYEGKYLMI---------HGDCVSIGCYAMTNQGIDEIFQFVTGALVFGQ
                    . :. **               :* **  :.  .    :     .

LdtD      ISD----ALKQGDTRYVNIRQSIPVNLY---------------YLTAFVG------AD--
LdtE      INEPVKYSVEPNGMRYVEVHRPLSAEEQQNVQTMPYTLPAGFTQFKDNKAVDQKL-----
LdtF      --PSVQVSIYPFRMTDANMKR----HKYSNFKDFWEQLKPGYDYFEQTRKPPTVSVVNGR
                 ::       .::::    .                  :

LdtD      --------GRTQYRTDIYNYDLPARSSSQIVSKAE-------QLIR---
LdtE      --VDKALYRRAGYPVSVSSGATPAASNAPSVESAQNGEPEQGNMLRVTQ
LdtF      YVVSKPLSHEVVQPQLASNYTLPEAK-----------------------
          ..   . * .
```

Figure S1. (**A**) Peptidoglycan cross-linking reactions catalyzed by the DD-transpeptidases (PBPs, top) and the LD-transpeptidases (LdtD, LdtE and LdtF, bottom) resulting in 4-3 and 3-3 cross-links, respectively. G, *N*-acetylglucosamine (G); M, *N*-acetylmuramic acid; R indicates H, D-Ala or D-Ala-D-Ala. (**B**) Sequence alignment of LdtD, LdtE and LdtF. LdtD sequence starts at amino acid 201 of the protein sequence. Residues flanking the Cys residue (**C**) of catalytic site are indicated in bold. Consensus symbols: (*) indicates fully conserved residues; (:) indicates residues with high degree of conservation; (.) indicates residues with low degree of conservation. Alignment was performed with ClustaOmega (https://www.ebi.ac.uk/Tools/msa/clustalo/).
