# Supplementary material for: Peptidoglycan Remodeling Enables Escherichia coli To Survive Severe Outer Membrane Assembly Defect
Source: mBio. 2019 Feb 5;10(1):e02729-18. doi: 10.1128/mBio.02729-18 (PMC6428754; doi:10.1128/mBio.02729-18)
Supplement: TABLE S1 [file mBio.02729-18-st001.docx]

| **Supplementary Table 1. Bacterial Strains and Plasmids.** | | |
| --- | --- | --- |
| **Strain or**  **Plasmid** | **Relevant Genotype, Features**  **or Characteristics** | **Source or Reference** |
| **Strain** |  |  |
| AMM05 | BW25113 ∆*ldtD*::frt | This work |
| AMM06 | BW25113 ∆*ldtE*::frt | This work |
| AMM07 | BW25113 ∆*ldtE*::frt ∆l*dtD*::frt | This work |
| AMM10 | BB-3 ∆*ldtD*::frt | This work |
| AMM11 | BB-3 ∆*ldtE*::frt | This work |
| AMM12 | BB-3 ∆*ldtD*::frt ∆*ldtE*::frt | This work |
| AMM14 | BB-3 ∆*ldtA*::frt ∆*ldtB*::frt ∆*ldtC*::frt | This work |
| AMM24 | BW25113 ∆*ldtF*::frt | This work |
| AMM25 | BW25113 ∆*ldtD*::frt ∆*ldtF*::frt | This work |
| AMM26 | BW25113 ∆*ldtE*::frt ∆*ldtF*::frt | This work |
| AMM28 | BW25113 ∆*ldtD*::frt ∆*ldtE*::frt ∆*ldtF*::frt | This work |
| AMM30 | BB-3 ∆*ldtF*::frt | This work |
| AMM31 | BB-3 ∆*ldtD*::frt ∆*ldtF*::frt | This work |
| AMM32 | BB-3 ∆*ldtE*::frt ∆*ldtF*::frt | This work |
| AMM33 | BB-3 ∆*ldtA*::frt ∆*ldtB*::frt ∆*ldtC*::frt ∆*ldtD*::frt ∆*ldtE*::frt ∆*ldtF*::frt | This work |
| AMM34 | BB-3 ∆*ldtD*::frt ∆*ldtE*::frt ∆*ldtF*::frt | This work |
| AMM36 | BW25113 ∆*rpoS*:: frt | This work |
| AMM51 | BW25113 ∆*mrcA*::frt | This work |
| AMM52 | BW25113 ∆*mrcB*::frt | This work |
| AMM53 | BW25113 ∆*dacA*::frt | This work |
| AMM54 | BW25113 ∆*dacC*::frt | This work |
| AMM55 | BW25113 ∆*lpoB*::frt | This work |
| AMM56 | BW25113 ∆*cpoB*::frt | This work |
| AMM60 | BB-3 ∆*mrcA*::frt | This work |
| AMM61 | BB-3 ∆*mrcB*::frt | This work |
| AMM62 | BB-3 ∆*dacA*::frt | This work |
| AMM63 | BB-3 ∆*dacC*::frt | This work |
| AMM64 | BB-3 ∆*lpoB*::frt | This work |
| AMM65 | BB-3 ∆*cpoB*::frt | This work |
| AMM83 | BW25113 *ldtD-his*::frt | This work |
| AMM84 | BB-3 *ldtD-his* | This work |
| BB-3 | BW25113 Φ(*kan* *araC* *araB*p*lptC*)1 | (Sperandeo *et al*., 2006) |
| BL21(DE3) | F– *ompT hsdS*B(rB– mB–) *gal dcm* (DE3) | Novagen |
| BW25113 | *lacI^q^ rrnB*_T14_ Δ*lacZ*_WJ16_ *hsdR514* Δ*araBAD*_AH33_ Δ*rhaBAD*_LD78_ | (Datsenko and Wanner, 2000) |
| BW25113Δ6LDT | *lacI^q^ rrnB*_T14_ Δ*lacZ*_WJ16_ *hsdR514* Δ*araBAD*_AH33_ Δ*rhaBAD*_LD78_  *ΔycbB ΔerfK ΔycfS ΔybiS ΔynhG ΔyafK* | (Kuru *et al*., 2017) |
| DH5α | Δ(*argF*-*lac169*) φ80 *dlacZ58*(M15) *glnV44*(AS) λ^-^ *rfbD1* *gyrA96 recA1* *endA1 spoT1 thi-1* *hsdR17* | (Hanahan, 1983) |
| JW0732 | BW25113 ∆*cpoB*::*kan* | (Baba *et al*., 2006) |
| JW0803 | BW25113 ∆*ldtB*790::*kan* | (Baba *et al*., 2006) |
| JW0908 | BW25113 ∆*ldtD*742::*kan* | (Baba *et al*., 2006) |
| JW1668 | BW25113 ∆*ldtE*753::*kan* | (Baba *et al*., 2006) |
| JW1968 | BW25113 ∆*ldtA*761::*kan* | (Baba *et al*., 2006) |
| JW5820 | BW25113 ∆*ldtC*775::*kan* | (Baba *et al*., 2006) |
| JW3359 | BW25113 ∆*mrcA*::*kan* | (Baba *et al*., 2006) |
| JW0145 | BW25113 ∆*mrcB*::*kan* | (Baba *et al*., 2006) |
| JW5157 | BW25113 ∆*lpoB*::*kan* | (Baba *et al*., 2006) |
| JW0627 | BW25113 ∆*dacA*::*kan* | (Baba *et al*., 2006) |
| JW0823 | BW25113 ∆*dacC*::*kan* | (Baba *et al*., 2006) |
| JW5437 | BW25113 ∆*rpoS*::*kan* | (Baba *et al*., 2006) |
| LOBSTR-BL21(DE3) | F– *ompT hsdS*B(rB– mB–) *gal dcm* (DE3), carries genomically modified copies of *arnA* and *slyD* | Kerafast |
| **Plasmid** |  |  |
| pDACAhis | pET28a(+)derivative, for overexpression of His-PBP5 | (Potluri *et al*., 2010) |
| pET28a-dacC | pET28a-dacC^28-400^ | This work |
| pGS100 | pGZ119EH derivative, contains TIR sequence downstream of p*tac*, Cam^R^ | (Sperandeo *et al*., 2006) |
| pMN86 | pET21b-yebA^40-440^ | (Singh *et al*., 2012) |
| pMUCα | pJFK118EH derivative, for ectopic expression of PBP1B | U. Bertsche, W. Vollmer, unpublished |
| pMUCα(mut) | pJFK118EH derivative, for ectopic expression of PBP1B S510A | U. Bertsche, W. Vollmer, unpublished |
| pMUC TG(mut)α | pJFK118EH derivative, for ectopic expression of PBP1B E233Q | U. Bertsche, W. Vollmer, unpublished |
| pMUC TG(mut) α(mut) | pJFK118EH derivative, for ectopic expression of PBP1B S510A E233Q | U. Bertsche, W. Vollmer, unpublished |
| pKD4 | oriR; Amp^R^ Kan^R^; source of *kan* cassette | Datsenko and Wanner, 2000 |
| pRS415 | pBR322 derivative; harbors the entire *lac* operon without promoter; Amp^R^ | (Simons *et al*., 1987) |
| pRS415-p*ldtD* | pRS415 derivative; expresses LacZ from the *ldtD* promoter region | This work |
| pRS415-p*ldtE* | pRS415 derivative; expresses LacZ from the *ldtE* promoter region | This work |
| pRS415-p*ldtF* | pRS415 derivative; expresses LacZ from the *ldtF* promoter region | This work |
| pET28a His6*-ldtF* | pET28a derivative; expresses LdtF from the T7 promoter starting from amino acid 20 and fused at N-terminal with 6xHis tag | This work |
| pETMM82 *dsbC*-His6-*ldtD* | pETMM82 derivative; expresses LdtD fused at N-terminal with DsbC and a 6×His tag | (Hugonnet *et al*., 2016) |
| pJEH12(*ldtD*) | pACYC184 derivative; expresses LdtD under the IPTG-inducible *trc* promoter; Tet^R^ | (Hugonnet *et al*., 2016) |
| pAMS01(*ldtE*) | pACYC184 derivative; expresses LdtE under the IPTG-inducible trc promoter; Tet^R^ | This work |
| pAMS02(*ldtF*) | pACYC184 derivative; expresses LdtF under the IPTG-inducible *trc* promoter; Tet^R^ | This work |
| pSAV057 | ptrc99A derivative; contains weakened -35 promotor region (TTGACA-TTTACA); p15 origin; Cam^R^ | (Alexeeva *et al*., 2010) |
| pGS121 | pGZ119H derivative; expresses LdtE under the *tac* promoter; Cam^R^ | This work |
| pGS123 | pGZ119H derivative; expresses LdtD under the *tac* promoter; Cam^R^ | This work |
| pGS123C528A | pGZ119H derivative; expresses LdtDC528A under the *tac* promoter; Cam^R^ | This work |
| pGS124 | pGZ119H derivative, expresses LdtF under the *tac* promoter; Cam^R^ | This work |
