# Supplementary material for: Peptidoglycan Remodeling Enables Escherichia coli To Survive Severe Outer Membrane Assembly Defect
Source: mBio. 2019 Feb 5;10(1):e02729-18. doi: 10.1128/mBio.02729-18 (PMC6428754; doi:10.1128/mBio.02729-18)
Supplement: FIG S2 [file mBio.02729-18-sf002.pdf]

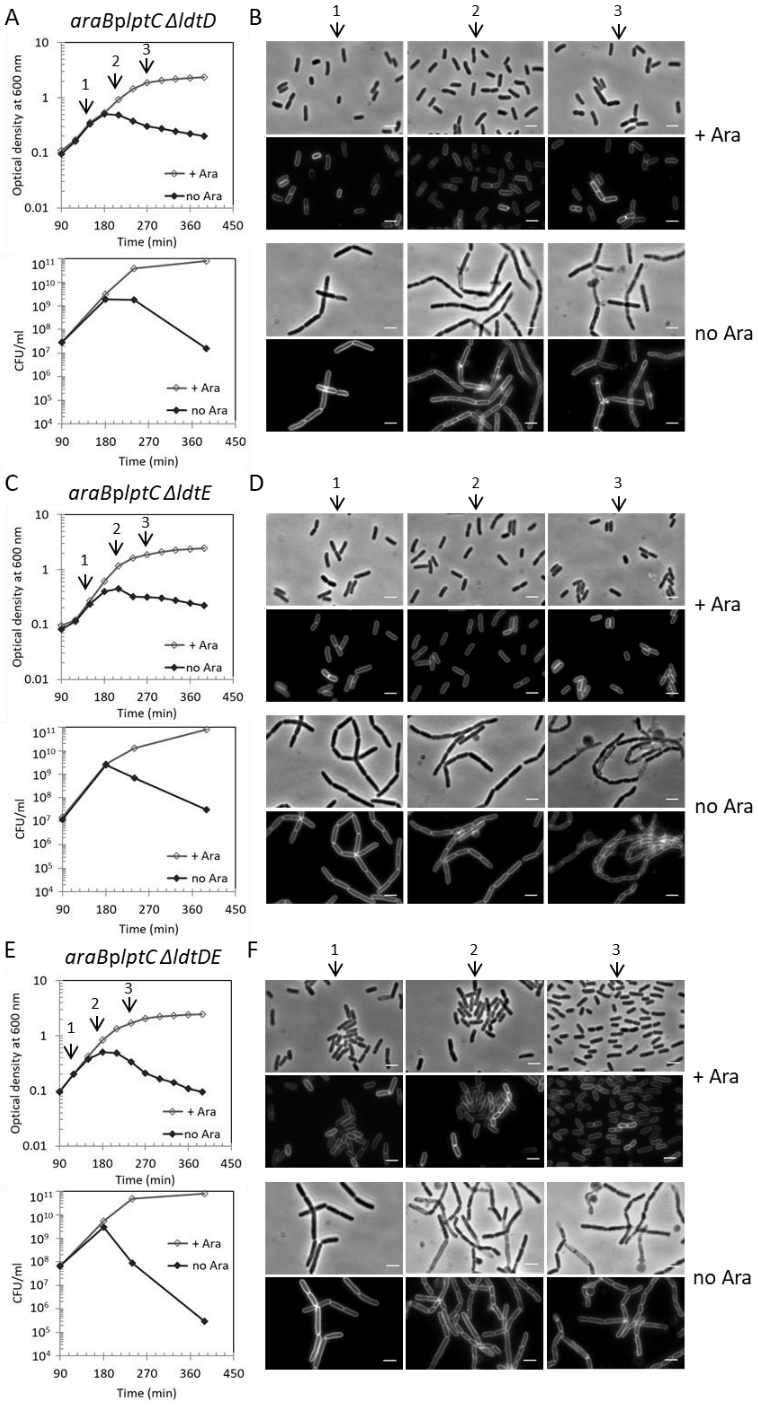

Figure S2. Deletion of *ldtD*, *ldtE* and *ldtD*-*ldtE* in the *araB*p*lptC* conditional strain compromises cell viability under non-permissive conditions. Cells of *araB*p*lptC* Δ*ldtD* (**A**), *araB*p*lptC* Δ*ldtE* (**C**) *araB*p*lptC* Δ*ldtD* Δ*ldtE* (**E**) were grown in the presence of 0.2% arabinose to an OD_{600} of 0.2, harvested, washed three times and resuspended in an arabinose-supplemented (+ Ara) or arabinose-free (no Ara) medium. Cell growth was monitored by OD_{600} measurements (upper panels) and viability was assessed by determining CFU (lower panels). Growth curves shown are representative of at least three independent experiments. At t = 120 min, 210 min and 270 min (arrows), a*raB*p*lptC* Δ*ldtD* (**B**), *araB*p*lptC* Δ*ldtE* (**D**) and *araB*p*lptC* Δ*ldtD* Δ*ldtE* (**F**) cells were collected for imaging. Phase contrast images are on the top and fluorescence images are on the bottom. Scale bars, 3 μm.
