# Supplementary material for: Peptidoglycan Remodeling Enables Escherichia coli To Survive Severe Outer Membrane Assembly Defect
Source: mBio. 2019 Feb 5;10(1):e02729-18. doi: 10.1128/mBio.02729-18 (PMC6428754; doi:10.1128/mBio.02729-18)
Supplement: FIG S3 [file mBio.02729-18-sf003.pdf]

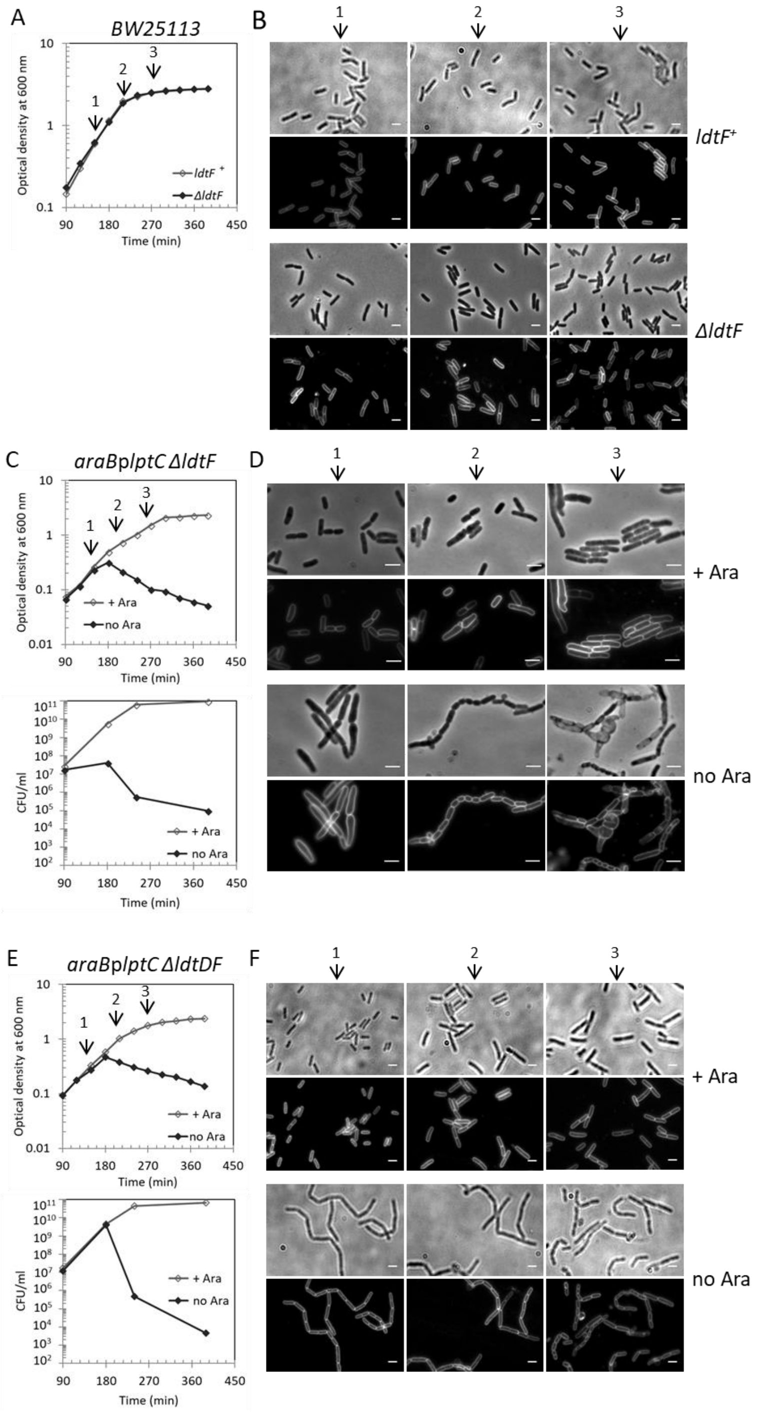

Figure S3. Phenotypes of wild type BW25113 (*lptC*$^+$) and *araB*p*lptC* conditional strains lacking *ldtF* and of *araB*p*lptC* lacking *ldtD*-*ldtF*. Cells of BW25113, the isogenic Δ*ldtF* mutant (**A**), the *araB*p*lptC* Δ*ldtF* mutant (**C**) and *araB*p*lptC* Δ*ldtD* Δ*ldtF* mutant (**E**) were grown and imaged as described in the legend of Figure S2. Growth curves shown are representative of at least three independent experiments. At t = 120 min, 210 min and 270 min (arrows), BW25113 and BW25113 Δ*ldtF* (**B**), *araB*p*lptC* Δ*ldtF* (**D**) *araB*p*lptC* Δ*ldtE* Δ*ldtF* mutant (**F**) cells were collected for imaging. Phase contrast images are on the top and fluorescence images are on the bottom. Scale bars 3 µm. *araB*p*lptC* Δ*ldtF* cells displayed morphological defects even when grown under permissive conditions.
