# Supplementary material for: Peptidoglycan Remodeling Enables Escherichia coli To Survive Severe Outer Membrane Assembly Defect
Source: mBio. 2019 Feb 5;10(1):e02729-18. doi: 10.1128/mBio.02729-18 (PMC6428754; doi:10.1128/mBio.02729-18)
Supplement: FIG S4 [file mBio.02729-18-sf004.pdf]

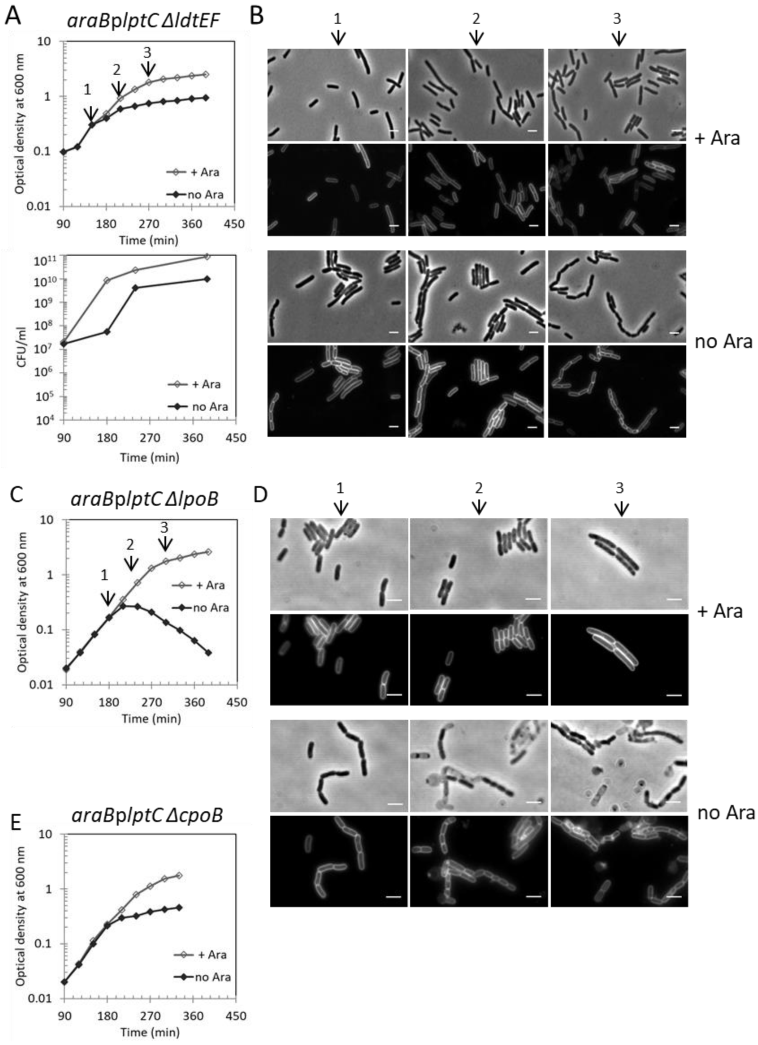

Figure S4. Growth profiles of *araB*p*lptC* conditional strain lacking *ldtE*-*ldtF* or *lpoB* or *cpoB.* Cells of *araB*p*lptC* Δ*ldtE* Δ*ldtF* (**A**, **B**), *araB*p*lptC* Δ*lpoB* (**C**, **D**) and *araB*p*lptC* Δ*cpoB* (**E**) were grown and imaged as described in the legend of Figure S2. Growth curves shown are representative of at least three independent experiments. *araB*p*lptC* Δ*ldtE* Δ*ldtF* cells did not lyse under non-permissive conditions (**A, B**). The PBP1B activator LpoB prevents lysis in *lptC*-depleted cells (**C, D**) whereas deletion of *cpoB* has no impact on cell viability (**E**).
