# Supplementary material for: Peptidoglycan Remodeling Enables Escherichia coli To Survive Severe Outer Membrane Assembly Defect
Source: mBio. 2019 Feb 5;10(1):e02729-18. doi: 10.1128/mBio.02729-18 (PMC6428754; doi:10.1128/mBio.02729-18)
Supplement: FIG S5 [file mBio.02729-18-sf005.pdf]

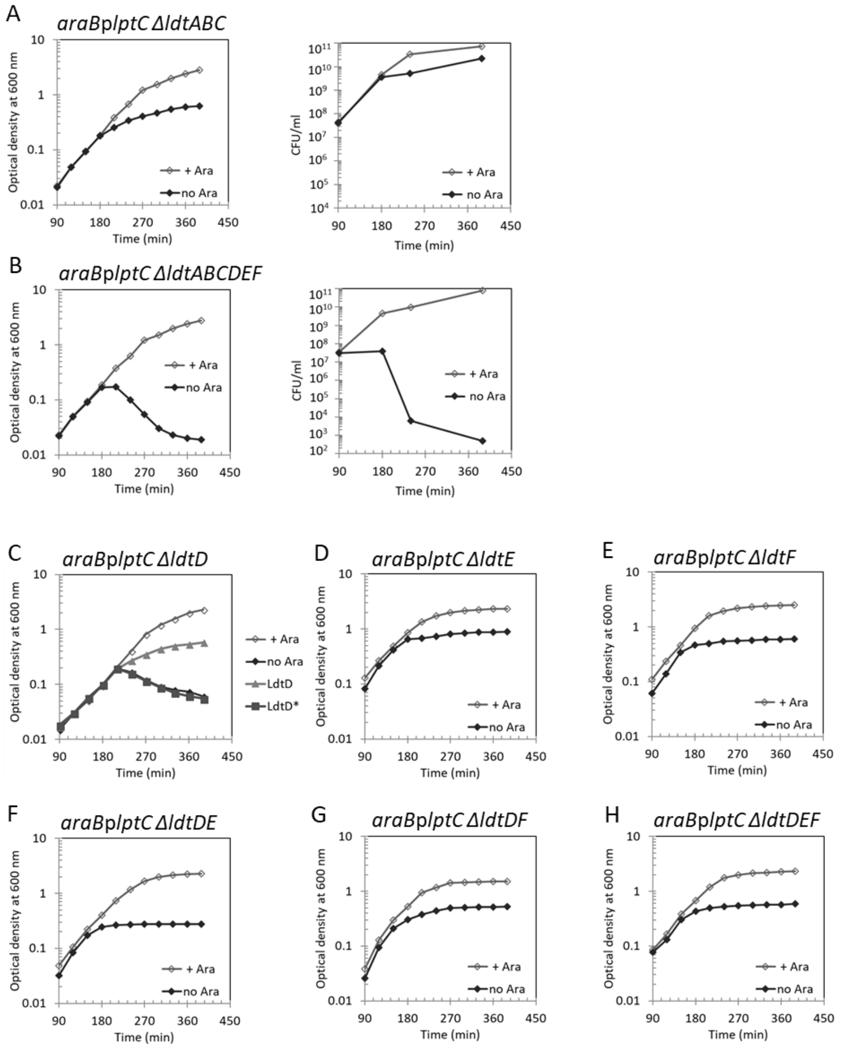

Figure S5. The simultaneous deletion of *ldtA*, *ldtB* and *ldtC* in the *araB*p*lptC* conditional strain has no impact on cell viability under non-permissive conditions and ectopic expression of LdtD rescues the lysis phenotype of single and multiple *araB*p*lptC ldt* mutants. Cells of the *araB*p*lptC* conditional strain with deletions of *ldtA*, *ldtB* and *ldtC* (**A**) and all six *ldt* genes (*ldtA*, *ldtB*, *ldtC*, *ldtD*, *ldtE* and *ldtF*) (**B**) were grown in the presence of 0.2% arabinose to an OD_{600} of 0.2, harvested, washed three times and resuspended in an arabinose-supplemented (+ Ara) or arabinose-free (no Ara) medium. Cell growth was then monitored by OD_{600} measurements and viability was assessed by determining the CFU. (**C**) Complementation of the *araB*p*lptC* Δ*ldtD* lysis phenotype by ectopic expression of wild type *ldtD* and $ldtD^{C528A}$. (**D-H**) Complementation of the *araB*p*lptC* Δ*ldtE, araB*p*lptC* Δ*ldtF*, *araB*p*lptC* Δ*ldtDE*, *araB*p*lptC* Δ*ldtDF*, *araB*p*lptC* Δ*ldtDEF* lysis phenotype by ectopic expression of wild type *ldtD.* Cells were grown in the presence of 0.2% arabinose to an OD_{600} of 0.2, harvested, washed three times and resuspended in an arabinose-free medium. The growth of *araB*p*lptC* Δ*ldtD* in arabinose-supplemented medium is shown as control. Cell growth was monitored by OD_{600} measurements. Growth curves shown are representative of at least three independent experiments. In panels A and B *ldt* genes are indicated by their capital letters.
