# Supplementary material for: Peptidoglycan Remodeling Enables Escherichia coli To Survive Severe Outer Membrane Assembly Defect
Source: mBio. 2019 Feb 5;10(1):e02729-18. doi: 10.1128/mBio.02729-18 (PMC6428754; doi:10.1128/mBio.02729-18)
Supplement: FIG S6 [file mBio.02729-18-sf006.pdf]

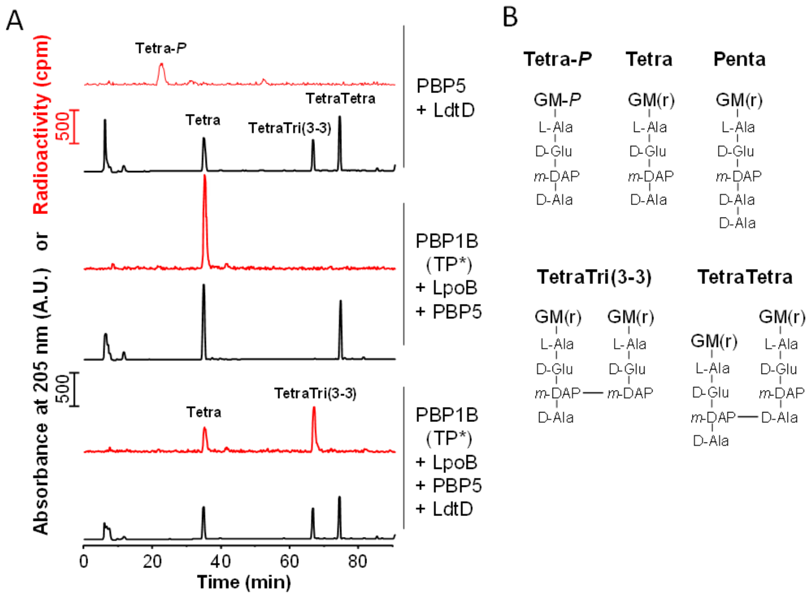

Figure S6. LdtD is active during *in vitro* PG synthesis in the presence of PBP1B(TP*), LpoB and PBP5. (**A**) HPLC chromatograms obtained from samples containing radioactive lipid II, PG from *E. coli* BW25113ΔLDT and the proteins indicated on the right side. (**B**) Proposed structures of muropeptides shown in panel A and B. G, *N*-acetylglucosamine; M, *N*-acetylmuramic acid; M(r), *N*-acetylmuramitol; M-*P*, N-acetylmuramic acid-1-phosphate; L-Ala, L-alanine; D-Glu, D-glutamic acid; D-Ala, D-alanine; *m*-DAP, *meso*-diaminopimelic acid.
