# Supplementary material for: Peptidoglycan Remodeling Enables Escherichia coli To Survive Severe Outer Membrane Assembly Defect
Source: mBio. 2019 Feb 5;10(1):e02729-18. doi: 10.1128/mBio.02729-18 (PMC6428754; doi:10.1128/mBio.02729-18)
Supplement: TABLE S2 [file mBio.02729-18-st002.docx]

| **Supplementary Table 2. Oligonucleotides.** | |  |  |
| --- | --- | --- | --- |
| **Primer** | **Sequence 5' -> 3'** | **Description** | **Used to make** |
| AP405/39 *ldtE*-f | CCGGAATTCACCATGAAACGCGCGTCTTTCCACTC | [CCG]-[*Eco*RI]-[ACC]-[start *ldtE*; fwd] | pGS121 construction |
| AP407/34 *ldtE*-r | CCCAAGCTTTTACTGCGTCACGCGTAACATATTC | [CCC]-[*Hind*III]-[TTA]-[stop *ldtE*; rev] | pGS121 construction |
| AP413/35 *ldtD*-f | ccggaattcaccATGTTGCTTAATATGATGTGTGG | [CCG]-[*Eco*RI]-[ACC]-[start *ldtD* fwd] | pGS123 construction |
| AP414/31-*ldtD*-r | ctagtctagaTTACCTGATTAATTGTTCCGC | [CTA]-[*Xba*I]-[stop *ldtD*  rev] | pGS123 construction |
| AP458/31 *ldtF*-f | CCGGAATTCATGCGTAAAATCGCATTAATTC | [CCG]-[*Eco*RI]-[start *ldtF*; fwd] | pGS124 construction |
| AP460/34 *ldtF*-r | CCCAAGCTTTTATTTTGCCTCGGGGAGCGTGTAG | [CCC]-[*Hind*III]-[TTA]-[stop *ldtF*; rev] | pGS124 construction |
| AP475/33 *ldtD*-f | CGAGAGGAATTCGTGGTACAAAGCTGGGAAGAT | [CGAGAG]-[*Eco*RI]-[579 bp upstream of ATG of *ldtD*; fwd] | *ldtD*p cloning in pRS416 |
| AP476/28 ldtD-r | CGCGGATCCTTTGCGTGCGGGCTTTTTC | [CGC]-[*Bam*HI]-[232 bp downstream of ATG of *ldtD*; rev] | *ldtD*p cloning in pRS416 |
| AP477/31 *ldtE*-f | CCGAGAGGAATTCGTATTCACCGTTTGCTGGG | [CCGAGAG]-[*Eco*RI]-[601 bp upsrteam of ATG of *ldtE*; fwd] | *ldtE*p cloning in pRS415 |
| AP478/27 *ldtE*-r | CGCGGATCCGGCGATAGTGTTATTGGC | [CGC]-[*Bam*HI]-[219 bp downstream of ATG of *ldtE*; rev] | *ldtE*p cloning in pRS415 |
| AP490/30 *ldtF*-f | GGAATTCCATATGGGTTTGCTGGGCAGCAG | [GGAATTC]-[*Nde*I]-[starting at 58bp downstream of ATG of *ldtF*; fwd] | pET28a His6-*ldtF* cloning |
| AP491/29 *ldt*F-r | CCGCTCGAGTTATTTTGCCTCGGGGAGCG | [CCG]-[*Xho*I]-[stop *ldtF*; rev] | pET28a His6-*ldtF* cloning |
| AP538/32 *ldtF*-f | CGAGAGGAATTCGAATCAGGCAGCGGACGTAC | [CGAGAG]-[*Eco*RI]-[614 bp upstream of ATG of *ldtF*; fwd] | *ldtF*p cloning in pRS415 |
| AP539/29 *ldtF*-r | TCCCCCCGGGCTCGCCCATTTTGACGTAG | [TCCC]-[*Sma*I]-[161 bp downstream of ATG of *ldtF*; rev] | *ldtF*p cloning in pRS416 |
| AP651/33-*ldtD*mut | GAGCTCAGGCgcaGTACGAGTGAATAAAGCTTC | [10 bp complement of *ldtD*]- [TGT>GCA substitution]-[20 bp complement to *ldtD*] | *ldtD* mutagenesis |
| AP652/18-*ldtD*r | AATGCGCGTGTATCACGC | [18 bp complement to *ldtD*] | *ldtD* mutagenesis |
| AP565/83-*ldtD*Chf | CCAGCTCGCAAATCGTATCGAAAGCGGAACAATTAATCAGGCATCATCATCATCATCATTAAgtgtaggctggagctgcttcg | [41 bp complement to *ldtD*]-[His tag coding sequence]-[taa stop codon]-[21 bp complement to *kan* cassette of pKD4] | *ldtD-his::kan* cassette construction |
| AP566/63-*ldtD*CHhr | CATGCTAATTATTACGACAACTGATTTCCCCGAACTACTTCATcatatgaatatcctccttag | [41 bp complement to downstream region of *ldtD*]-[20 bp complement to *kan* cassette of pKD4] | *ldtD-his::kan* cassette construction |
| Pbp6a-sp_for | GCGCGCCATATGGCGGAACAAACCGTTG | [GCGCGC]-[*Nde*I]-[starting at 82 bp downstream of ATG of *dacC*; fwd] | pET28a His6-*dacC* cloning |
| Pbp6a-sp_rev | GCGCGCCTCGAGTTAAGAGAACCAGCTGCC | [GCGCGC]-[*XhoI*]-[stop *dacC*; rev] | pET28a His6-*dacC* cloning |
| AMS-GA7h-F | ATTTCACACAGGAAACAGACCATGGATGAAACGCGCGTCTTTGCTTAC | [24 bp complement of pJEH12(*ldtD*)]-[start of *ldtE*; fwd] | pAMS01(*ldtE*) construction |
| AMS-GA7h-R | GCATGCCTGCAGGTCGACTCTAGACTACTGCGTCACGCGTAACATATTC | [24 bp complement of pJEH12(*ldtD*)]-[stop of *ldtE*; fwd] | pAMS01(*ldtE*) construction |
| AMS-GA7a_F | ATTTCACACAGGAAACAGACCATGGATGCGTAAAATCGCATTAATTC | [24 bp complement of pJEH12(*ldtD*)]-[start of *ldtF*; fwd] | pAMS02(*ldtF*) contruction |
| AMS-GA7a_R | GCATGCCTGCAGGTCGACTCTAGATTATTTTGCCTCGGGGAGCG | [24 bp complement of pJEH12(*ldtD*)]-[stop of *ldtF*; fwd] | pAMS02(*ldtF*) contruction |
| AMS-GA7_F | TCTAGAGTCGACCTGCAGGCATGC | pACYC plasmid linearization for Gibson; fwd | pAMS01(*ldtE*) and pAMS2(*ldtF*) contruction |
| AMS-GA7_R | CCATGGTCTGTTTCCTGTGTGAAATT | pACYC plasmid linearization for Gibson; rev | pAMS01(*ldtE*) and pAMS2(*ldtF*) contruction |
