# Supplementary material for: Peptidoglycan Remodeling Enables Escherichia coli To Survive Severe Outer Membrane Assembly Defect
Source: mBio. 2019 Feb 5;10(1):e02729-18. doi: 10.1128/mBio.02729-18 (PMC6428754; doi:10.1128/mBio.02729-18)
Supplement: TEXT S1 [file mBio.02729-18-s0001.pdf]

## Supplemental Methods

### Construction of *E. coli* deletion or depletion strains

Deletion strains were obtained by moving *kan*-marked alleles from the Keio *E. coli* single-gene knockout library (1) by P1 phage transduction (2). Afterward, the *kan* cassette was removed by pCP20-encoded Flp recombinase to generate unmarked deletions with a FRT-site scar sequence (3). The removal of the *kan* gene was verified by colony PCR. Strains with multiple deletions were generated by sequential P1 transduction and *kan* cassette removal. *LptC* depletion strains were obtained by moving the *kan araC araB*p-*lptC* allele from BB-3 (4) into selected mutants by P1 transduction. Depletion strains were selected on media containing kanamycin and 0.2% arabinose. The insertion of the cassette was verified by PCR.

### Construction of *ldtD*-his strains

The λ Red recombination system was used to fuse the His-tag coding sequence to chromosomally encoded *ldtD* to produce a *ldtD-his* C-terminal fusion. A linear PCR product encoding the *his*-tag and the *kan* cassette flanked by sequences for homologous recombination (Table S1) was obtained using primers AP565 and AP566 and as a template pKD4 (Table S2). and transformed into BW25113 strain harbouring the pKD46, a Red helper plasmid (3). The correct insertion of the *his-kan* cassette was confirmed by PCR. Afterward, the *kan* cassette was removed as described above. The *araB*p-*lptC ldtD-his* strain was obtained by moving the *kan araC araB*p-*lptC* allele from BB-3 (4) as described above.

**Construction of plasmids**

pGS121 and pGS124 were constructed by cloning *ldtE* and *ldtF* into the *Eco*RI/*Hind*III restriction sites of pGS100 (4). pGS123 was constructed by cloning *ldtD* into *Eco*RI/*Xba*I restriction sites of pGS100. Primers used for genes cloning are listed in Table S2. To assess transcriptional activity the promoter regions of *ldtE*, *ldtD* and *ldtF* genes were cloned into the *lacZ* vector pRS415 (5). For this, the promoter region of each *ldt* gene was amplified by PCR using primers listed in Table S2 and cloned into *EcoR*I/*Bam*HI (*ldtE*p and *ldtD*p) or *EcoR*I/*Sma*I (*ldtF*p) restriction sites of pRS415. Each cloned region contained at least 600 bp upstream and 150 bp downstream of the start codon of each gene to include putative regulatory elements.

For pET28a-His6-LdtF, *ldtF* was cloned starting from position 58 downstream the ATG codon, into *Nde*I/*Xho*I pET28a, eliminating the putative signal sequence. The correct nucleotide sequences of inserts were verified (Eurofins Genomics).

pAMS01(LdtE) and pAMS02(LdtF) were constructed using the Gibson assembly method (6) by cloning *ldtE* and *ldtF* into pJEH12(LdtD) (7), respectively. Primers used for gene cloning are listed in Table S2.

pGS123C528A was obtained by site-directed mutagenesis using the Q5® Site-Directed Mutagenesis Kit (New England BioLabs) on pGS123 following manufacturer instructions. Primers used for site directed mutagenesis are listed in Table S2.

**Measurements of the minimum inhibitory concentration (MIC) of LPC-058.**

The MIC assay protocol was adapted from (8) using 96-well plates. Bacterial were grown in LB-Lennox medium at 37°C in the presence of varying concentration of LPC-058 inhibitor and 5% of DMSO as control.

## Overexpression plasmids and purification of proteins

**Purification of PBP6a**. DNA encoding for PBP6a (residues 28-400) from *E. coli* BW25113 was amplified by PCR and cloned into pET28a(+) using *Nde*I and *Xho*I. PBP6a was overexpressed in *E. coli* LOBSTR-BL21(DE3) (Kerafast) cells grown overnight at 37°C in 2 L of TB-autoinduction medium supplemented with 4 g lactose, 1 g glucose, 10 mM $MgCl_2$ and 10 mM $MgSO_4$ (9). Cells were harvested by centrifugation for 15 min at 4500 rpm and 14°C. The resulting cell pellet was resuspended in 50 ml buffer A (25 mM HEPES/NaOH pH 7.5, 100 mM NaCl) supplemented with 1 mM phenylmethyl sulfonyl fluoride (Sigma Aldrich), 1× protease inhibitor cocktail (Sigma Aldrich) and desoxyribonuclease I (Sigma Aldrich). Cells were broken by sonication and centrifuged for 1 h at 130,000×*g* and 4 °C. The resulting pellet was resuspended in buffer B (25 mM HEPES/NaOH pH 7.5, 1 M NaCl, 2 mM $MgCl_2$ 10% glycerol) supplemented with 1% CHAPS (Anatrace) and incubated under continuous stirring overnight at 4°C. Insoluble material was removed by centrifugation for 1 h at 130,000×*g* at 4°C. The supernatant was recovered, mixed with 1 ml Ni-NTA Superflow (Qiagen) preequilibrated in buffer B (supplemented with 0.5% CHAPS and 5 mM imidazole) and incubated under continuous gentle stirring for 3 h at 4°C. Ni-NTA agarose was poured in a gravity flow column, washed 5 times with 20 column volume (CV) buffer B (supplemented with 0.5% CHAPS and increasing concentrations of imidazole, 10-50 mM). PBP6a was eluted with buffer B supplemented with 0.5% CHAPS and 300 mM imidazole. Eluted protein was dialysed against 2 L dialysis buffer (25 mM HEPES/NaOH, 500 mM NaCl, 10% glycerol, 0.1% CHAPS, 10 mM EDTA). The protein was further purified by size exclusion chromatography on a HiLoad 16/60 Superdex 200 (GE Healthcare) column using size exclusion buffer (25 mM HEPES/NaOH, 300 mM NaCl, 10% glycerol, 0.1% CHAPS) and a flowrate of 1

ml/min. Purity was determined by SDS-PAGE and combined fractions were concentrated and stored in aliquots at -80°C.

**Purification of LdtD.** *E. coli* LOBSTR-BL21(DE3) (Kerafast) cells were transformed with pETMM82, a plasmid encoding for LdtD carrying an N-terminal DsbC-His6-tag followed by a TEV-protease cleavage site (7), and grown at 30°C in 1 L TB medium (10) (supplemented with 5 mM $MgCl_2$ and 5 mM $MgSO_4$) until OD600 0.3. LdtD overexpression was induced by adding IPTG (Generon) to a final concentration of 0.5 mM. The temperature was decreased to 16°C and cells were incubated for 19 h. Cells were harvested by centrifugation for 15 min at 4,500 rpm and 14°C. The resulting cell pellet was resuspended in 60 ml buffer A (20 mM Tris pH 8.0, 1 M NaCl, 10 mM imidazole) supplemented with 1 mM phenylmethyl sulfonyl fluoride (Sigma Aldrich), 1× protease inhibitor cocktail (Sigma Aldrich) and desoxyribonuclease I (Sigma Aldrich). Cells were broken by sonication and centrifuged for 1 h at 130,000×*g* at 4°C. The supernatant was recovered, mixed with 0.5 ml Ni-NTA Superflow (Qiagen) preequilibrated in buffer A (supplemented with 10 mM imidazole) and incubated under continuous gentle stirring at 4°C. After 1.5 h another 0.5 ml of Ni-NTA Superflow (Qiagen) was added and incubated for 1.5 h. The suspension was poured in a gravity flow column and washed 2 times with 20 CV buffer B (20 mM Tris/HCl pH 7.0, 150 mM NaCl) supplemented with 20 mM imidazole, 5 mM ATP and 1 mM $MgCl_2$ to remove tightly bound chaperone proteins. After 3 more washing steps with 20 CV of buffer B each (2× 40 mM imidazole, 1× 50 mM imidazole), the protein was eluted with buffer B supplemented with 300 mM imidazole and glycerol was added to the elution fractions to a final concentration of 10%. The protein was dialysed against 2× 2 L dialysis buffer 1 (25 mM Tris pH 7.0, 300 mM NaCl, 10% glycerol) for 1 h each at

4°C. The protein solution was supplemented with 5 mM β-mercaptoethanol (Sigma Aldrich), 10 U/ml TEV-protease (Promega) and dialysed against 1 L of dialysis buffer 2 (25 mM Tris pH 7.0, 300 mM NaCl, 5 mM β-mercaptoethanol, 10% glycerol) for 1 h and against an additional 1 L overnight at 4°C.

The sample was mixed with 1 ml of Ni-NTA-agarose preequilibrated in dialysis buffer 2 containing 50 mM of imidazole and incubated for 2-3 h at 4°C under gentle stirring. The suspension was poured in a gravity flow column and the DsbC-His-tag free protein present in the flow through was further purified by size exclusion chromatography on a HiLoad 26/60 Supedex 200 (GE Healthcare) column using size exclusion buffer (25 mM Tris/HCl pH 7.5, 300 mM NaCl, 10% glycerol) and a flowrate of 1 ml/min. Purity was determined by SDS-PAGE and combined fractions were concentrated and stored in aliquots at -80°C.

**Purification of His-PBP1A and PBP1A.** PBP1A was purified according to a published procedure (11) with modifications. *E. coli* LOBSTR-BL21(DE3) (Kerafast) cells carrying the plasmid pTK1Ahis were grown in 2 L of LB medium (12) at 30°C until an optical density (578 nm) of 0.5 was reached. IPTG (1 mM) was added and the cells were grown for 3 h, chilled on ice for 15 min, harvested by centrifugation for 20 min at 5,000 rpm and $4^{0}$C. The cell pellet was resuspended in 140 ml of Buffer I (25 mM HEPES/NaOH pH 7.5, 1 M NaCl, 1 mM EGTA, 10% glycerol) supplemented with 1 mM phenylmethyl sulfonyl fluoride (Sigma Aldrich), 1× protease inhibitor cocktail (Sigma Aldrich) and desoxyribonuclease I (Sigma Aldrich). Cells were broken by sonication and the soluble fraction was removed after ultracentrifugation for 1 h at 130,000×*g* and 4°C. The membrane pellet was resuspended in extraction buffer (25 mM HEPES/NaOH pH 7.5, 5 mM $MgCl_2$, 1 M NaCl, 20% glycerol, 2% Triton X-100)

with continuous stirring overnight at 4°C. Insoluble material was removed by centrifugation for 1 h at 130,000×*g* at 4°C. The supernatant containing the solubilised membrane fraction was diluted with the same volume of IMAC dilution buffer (25 mM HEPES/NaOH pH 7.5, 1 M NaCl, 40 mM imidazole, 20% glycerol) and applied to a 5 mL HisTrap HP column using an ÄKTA PrimePlus. The column was washed with IMAC wash buffer (25 mM HEPES/NaOH pH 7.5, 500 mM NaCl, 50 mM imidazole, 20% glycerol, 0.2% reduced Triton X-100), and PBP1A was eluted with the elution buffer (25 mM HEPES/NaOH pH 7.5, 500 mM NaCl, 400 mM imidazole, 20% glycerol, 0.2% reduced Triton X-100). Fractions containing His-PBP1A were pooled. For the removal of the His-tag, 16 units of thrombin (restriction grade, Novagen) were added and the sample was dialysed in 3 × 1 l of cleavage buffer (20 mM HEPES/NaOH pH 7.5, 500 mM NaCl, 10% glycerol).

**Purification of MepM.** The protein was purified as described in (13) with modifications. Briefly, 2 L of LB medium (12) were inoculated with strain BL21(DE3) pET21b-*yebA* and protein expression was induced by addition of IPTG at a final concentration of 50 µM. Cells were incubated for 2 h at 25°C, harvested by centrifugation and resuspended in lysis buffer (25 mM Tris/HCl pH 7.5, 300 mM NaCl, 10% glycerol). The first purification step was performed on HisTrap HP column (GE healthcare) preequilibrated with wash buffer (25 mM Tris/HCl pH 7.5, 300 mM NaCl, 20 mM imidazole). Protein was eluted in the same buffer supplemented with 300 mM imidazole. Samples containing protein of interest were dialysed against 25 mM HEPES/NaOH pH 7.5, 300 mM NaCl, 10% glycerol overnight at 4°C. Dialysed samples were concentrated using Vivaspin 6 columns and applied to a HiLoad 16/60

Superdex 200 (GE healthcare) size exclusion column at a flowrate of 1 ml/min using the same buffer. The purified protein was stored in aliquots at -80 °C.

**Other proteins.** PBP1B and PBP1B(TP*) were purified as described in (14), LpoB was purified as described in (15), PBP5 was purified as described in (16).

**Protein-protein interactions**

Pull-down experiments were performed as described (17) using proteins at 2 μM concentration. Microscale thermophoresis (MST) experiments were carried out with a Monolith NT.115 instrument (NanoTemper GmbH, Germany). LdtD was labelled with the Monolith NT.115 Protein Labelling Kit RED-NHS according to the manufactory instructions. MST experiments were performed with serial dilution series of PBP1A or PBP1B and constant concentration of labelled LdtD in 20 mM HEPES/NaOH pH 7.5, 150 mM NaCl, 0.2 % Triton X-100, using premium capillaries, an LED-Power of 20% and an MST-Power of 40%. Changes in normalised fluorescence caused by the local temperature gradient were analysed by the MO.Affinity Analysis v2.1.2 software.

***In vivo* DTSSP cross-linking**

800 OD of BW25113 *ldtD*-his and *araB*p*lptC ldtD*-his cells were pelleted by centrifugation and resuspended in 25-mL ice-cold CL Buffer I (50 mM $NaH_2PO_4$, 20% [wt/vol] sucrose, pH 7.4) with 100 μg/mL DTSSP (freshly prepared as a 20 mg/ml stock in CL Buffer I). Cells were incubated at 4°C with mixing for 1 h, then pelleted and frozen at −80°C (17). Cells were then thawed, resuspended in 20 ml of 50 mM potassium phosphate buffer (pH 8.0), 150mM NaCl, 5mM $MgCl_2$, DNase I (50 μg/mL), and RNase I (50 μg/mL) and then lysed by sonication (3× 30 s at 20 mA). The lysate

was incubated with 1% ZW3-14 (*N*-tetradecyl-*N*,*N*-dimethyl-3-ammonio-1-propanesulfonate) for 20 min at room temperature with shaking to complete cell lysis. The mixture was then centrifuged at 10,000 *g* for 10 min to remove cell debris. To the cleared lysate (whole-cell extract) 20 mM imidazole (pH 8.0) was added, and the final mixture was loaded onto a 0.5-ml Ni-NTA column. Protein were eluted as described (18) and equal amount of proteins were used for Western blot analysis using Monoclonal Anti-polyHistidine antibody, anti-PBP1B (15) and anti-LptE (kind gift of D. Kahne) as loading control.

***In vivo* DTSSP cross-linking / co-immunoprecipitation assay**

The assay was performed as described in[17]. *araB*p*lptC ldtD-his* cells were grown overnight in LB Lennox media supplemented with 50 µg/mL kan and 0.2% L-arabinose, diluted 1:500 into 200 mL LB Lennox media with the same supplements. The cells were grown at 30°C to an $OD_{578}$ of 0.2, washed 3 × with LB Lennox media (no supplements; centrifugation at 1500 × *g*, 10 min at RT), resuspended in 200 mL Lennox LB (supplemented with 50 µg/mL kan) until the cells arrested growth. Cells were pelleted by centrifugation (1500 × *g*, 4°C, 10 min) and resuspended in 6 mL of ice-cold CL Buffer I (50 mM $NaH_2PO_4$ 20% (w/v) sucrose pH 7.4) with 100 µg/mL DTSSP (freshly prepared from a 20 mg/mL stock in CL Buffer I). Cells were incubated at 4°C with mixing for 1 h, then pelleted and resuspended to an $OD_{578}$ of 4 in ice-cold CL Buffer II (100 mM Tris/HCl, 10 mM $MgCl_2$, 1 M NaCl, pH 7.5) with 100 µM PMSF, 50 µg/mL protease inhibitor cocktail (P8465, Sigma-Aldrich) and 50 µg/mL DNAse I. The cells were disrupted by sonication and membranes were sedimented by centrifugation (90,000 × *g*, 60 min, 4°C) and resuspended in CL buffer III (25 mM Tris/HCl, 10 mM $MgCl_2$, 1 M NaCl, 20% glycerol, 2% Triton X-100, pH 7.5).

Membrane proetins were extracted by stirring overnight at 6°C. After another centrifugation step (90,000 × g, 1 h, 4°C) the supernatant was taken and 3-fold diluted with CL buffer IV (75 mM Tris/HCl, 10 mM $MgCl_2$, 1 M NaCl, pH 7.5). Specific PBP1B antibodies were added and the sample was incubated for 5 h at 4°C. A control sample was incubated without antibody. Protein G-coupled agarose (120 µl suspension) was added to the membrane fraction and the sample was incubated overnight at 4°C. The beads were recovered by centrifugation and washed with 10 ml of CL wash buffer (42 mM Tris/HCl, 10 mM $MgCl_2$, 1 M NaCl, 0.7% Triton X-100, 13% glycerol, pH 7.5) and boiled for 10 min in 50 µl of sample buffer for SDS-PAGE. The supernatant was collected, proteins were separated by SDS-PAGE and transferred to nitrocellulose by Western Blot, and LdtD-his was detected with a monoclonal anti-polyhistidine antibody (Sigma No. H1029) and an anti-mouse IgA - peroxidase antibody (Sigma No. A4789).

**MepM digest of sacculi from BW25113Δ6LDT**

Sacculi from BW25113Δ6LDT were prepared as described in (19). MepM digest was carried out in a final volume of 200 µl containing 25 mM HEPES/NaOH pH 7.5, 150 mM NaCl, 0.05% Triton X-100, 750 µg sacculi using a final concentration of MepM of 2 µM. The sample was incubated overnight at 37°C. Then the reaction mixture was heated for 10 min at 100°C and centrifuged for 20 min. The supernatant containing disaccharide-tetrapeptide chains was collected and stored at 2-8°C.

**LdtD activity assay with disaccharide-tetrapeptide chains or PG sacculi**

Assays were carried out in a final volume of 50 µl containing 25 mM Tris/HCl pH 7.5, 100 mM NaCl, 10 mM $MgCl_2$, 0.1% Triton X-100, and 2 µM LdtD. Fifteen µl of

peptidoglycan or 20 µl of disaccharide-tetrapeptide chains were added and the reaction mixture was incubated at 37°C overnight. The reaction was stopped by boiling the samples for 10 min.

**Coupled PG synthesis - LDT assay**

Coupled assays were carried out in a final volume of 50 µl containing 25 mM HEPES/NaOH pH 7.5, 175 mM NaCl, 10 mM $MgCl_2$, 0.1% Triton X-100, [$^{14}$C]Glc*N*Ac-labelled lipid II [10,000 dpm; (21)], 15 µl of PG from BW25113Δ6LDT and 2 µM of each protein as needed (LdtD, PBP1B-TP*, LpoB, PBP6 and/or PBP5). The reaction mixture was incubated for 4 h at 37°C. The reaction was stopped by boiling the samples for 10 min.

**LDTs expression for HPLC analysis**

BW25113Δ6LDT strain was transformed with pJEH12(LdtD), pAMS01(LdtE), pAMS02(LdtF) or an empty plasmid (pSAV057;[20) . Empty BW25113Δ6LDT was used as control. The same strain was also co-transformed with pJEH12(LdtD) and pGS124 or pAMS02(LdtF) and pGS121. A single transformant was used to inoculate 5 mL of Antibiotic Broth (AB) (Sigma Aldrich) overnight at 37°C. A 1:1000 dilution was performed in fresh AB cultures (400 mL each, in duplicate) from the overnight cultures. Samples were grown at 37°C and expression of LDTs was carried out with 50 µM IPTG when $OD_{600}$ was 0.2. After reaching the late exponential phase ($OD_{600}$ 0.8), samples were cooled in ice and harvested by centrifugation at 4°C. The cell pellet was resuspended in 6 ml ice-cold water and dropped slowly into 6 ml boiling 8% SDS water solution. Samples were boiled for 60 minutes.

## HPLC analysis

Samples were centrifuged for 20 minutes and the supernatant recovered and adjusted to pH 4 with 20% phosphoric acid. HPLC analysis was carried out as described in (21). Muropeptides were detected by online radioactivity detector and absorbance at 205 nm.

## Imaging and image analysis

Microscopy images were obtained with a Nikon Eclipse Ti microscope through a 100× 1.45 oil objective and photometric/Cool-SNAP-HQ2 camera or with a Zeiss Axiovert 200M microscope through a 63× 1.45 oil objective coupled to a AxioCam Mrm device 290 camera (Zeiss). Cells at different time points, as indicated by arrows in the figures, were collected from a total amount corresponding to an OD of 4, and a 1:10 ratio of fixation solution (fixation solution: formaldehyde 37% - glutaraldehyde 25% in PBS) was added. Cells were incubated for 30 min at 37°C with shaking, washed with PBS and resuspended in 500 µl of PBS. A cell suspension (5 µl) was spotted onto an agarose-coated glass slide (1% agarose), the sample was covered with a glass coverslip. To stain cell membranes, SynaptoRed C2M or FM5-95 was added to agarose solution to a final concentration of 2 µg/ml.

## β-galactosidase assay

β-galactosidase specific activity was measured from a total number of cells corresponding to an $OD_{600}$ of 8 as previously described (22).

## References

1. Baba T, Ara T, Hasegawa M, Takai Y, Okumura Y, Baba M, Datsenko, KA, Tomita M, Wanner BL, and Mori H. 2006. Construction of *Escherichia coli* K-12 in-frame, single-gene knockout mutants: the Keio collection. Mol Syst Biol *2*, 2006 0008

2. Silhavy TJ, Berman ML, and Enquist LW. 1984. Experiments with gene fusions., C.S.H. ColdSpringHarborLaboratory N.Y. , Editor.

3. Datsenko KA, and Wanner, BL. 2000. One-step inactivation of chromosomal genes in *Escherichia coli* K-12 using PCR products. Proc Natl Acad Sci U S A *97*, 6640-6645

4. Sperandeo P, Pozzi C, Dehò G, and Polissi A. 2006. Non-essential KDO biosynthesis and new essential cell envelope biogenesis genes in the *Escherichia coli yrbG-yhbG* locus. Res Microbiol *157*, 547-558.

5. Simons RW, Houman F, and Kleckner N. 1987. Improved single and multicopy *lac*-based cloning vectors for protein and operon fusions. Gene *53*, 85-96.

6. Gibson DG, Young L, Chuang RY, Venter JC, Hutchison CA 3rd, and Smith HO. 2009. Enzymatic assembly of DNA molecules up to several hundred kilobases. Nat Methods *6*, 343-345.

7. Hugonnet JE, Mengin-Lecreulx D, Monton A, den Blaauwen T, Carbonnelle E, Veckerle C, Brun YV, van Nieuwenhze M, Bouchier C, Tu K, Rice LB, Arthur M. 2016. Factors essential for L,D-transpeptidase-mediated peptidoglycan cross-linking and beta-lactam resistance in *Escherichia coli*. Elife 5:pii: e19469

8. Wiegand I, Hilpert K, Hancock RE. 2008. Agar and broth dilution methods to determine the minimal inhibitory concentration (MIC) of antimicrobial substances Nat Protoc. *3*,163-75. doi: 10.1038/nprot.2007.521

9. Studier FW. 2005. Protein production by auto-induction in high density shaking cultures. Protein Expr Purif *41*, 207-234.

10. Tartof KDH, CA. 1987. Improved media for growing plasmid and cosmid clones. Bethesda Res Lab Focus 9.

11. Born P, Breukink E, Vollmer W. 2006. In vitro synthesis of cross-linked murein and its attachment to sacculi by PBP1A from *Escherichia coli*. J Biol Chem 281:26985-93.

12. Miller JH. 1972. Experiments in molecular genetics. Cold Spring Harbor Laboratory, Cold Spring Harbor, New York.

13. Singh SK, SaiSree L, Amrutha RN, and Reddy M. 2012. Three redundant murein endopeptidases catalyse an essential cleavage step in peptidoglycan synthesis of *Escherichia coli* K12. Mol Microbiol 86, 1036-1051.

14. Typas A, Banzhaf M, van den Berg van Saparoea B, Verheul J, Biboy J, Nichols RJ, Zietek M, Beilharz K, Kannenberg K, von Rechenberg M, Breukink E, den Blaauwen T, Gross CA, Vollmer W. 2010. Regulation of peptidoglycan synthesis by outer-membrane proteins. Cell 143:1097-109.

15. Egan AJ, Jean NL, Koumoutsi A, Bougault CM, Biboy J, Sassine J, Solovyova AS, Breukink E, Typas A, Vollmer W, Simorre JP. 2014. Outer-membrane lipoprotein LpoB spans the periplasm to stimulate the peptidoglycan synthase PBP1B. Proc Natl Acad Sci U S A 111:8197-202.

16. Peters K, Kannan S, Rao VA, Biboy J, Vollmer D, Erickson SW, Lewis RJ, Young KD, Vollmer W. 2016. The Redundancy of Peptidoglycan Carboxypeptidases Ensures Robust Cell Shape Maintenance in *Escherichia coli*. MBio 7 e00819-16.
17. Gray AN, Egan AJ, Van't Veer IL, Verheul J, Colavin A, Koumoutsi A, Biboy J, Altelaar AF, Damen MJ, Huang KC, Simorre JP, Breukink E, den Blaauwen T, Typas A, Gross CA, Vollmer W. 2015. Coordination of peptidoglycan synthesis and outer membrane constriction during *Escherichia coli* cell division. Elife 4 e07118
18. Sperandeo P, Villa R, Martorana AM, Samalikova M, Grandori R, Dehò G, Polissi A. 2011. New insights into the Lpt machinery for lipopolysaccharide transport to the cell surface: LptA-LptC interaction and LptA stability as sensors of a properly assembled transenvelope complex. J Bacteriol 193:1042-53.
19. Glauner B, Holtje JV, Schwarz U. 1988. The composition of the murein of *Escherichia coli*. J Biol Chem 263:10088-95.
20. Alexeeva S, Gadella TW Jr., Verheul J, Verhoeven GS, and den Blaauwen T. 2010. Direct interactions of early and late assembling division proteins in *Escherichia coli* cells resolved by FRET. Mol Microbiol 77, 384-398.
21. Bertsche U, Breukink E, Kast T, Vollmer W. 2005. *In vitro* murein peptidoglycan synthesis by dimers of the bifunctional transglycosylase-transpeptidase PBP1B from *Escherichia coli.* J Biol Chem 280:38096-101.
22. Martorana AM, Sperandeo P, Polissi A, and Deho G. 2011. Complex transcriptional organization regulates an *Escherichia coli* locus implicated in lipopolysaccharide biogenesis. Res Microbiol 162, 470-482.
